# Biotinylated Polymer-Ruthenium Conjugates: In Vitro and In Vivo Studies in a Triple-Negative Breast Cancer Model

**DOI:** 10.3390/pharmaceutics14071388

**Published:** 2022-06-30

**Authors:** Leonor Côrte-Real, Ana Rita Brás, Adhan Pilon, Nuno Mendes, Ana Sofia Ribeiro, Tiago D. Martins, José Paulo S. Farinha, M. Conceição Oliveira, Fátima Gärtner, M. Helena Garcia, Ana Preto, Andreia Valente

**Affiliations:** 1 Centro de Química Estrutural, Institute of Molecular Sciences and Departamento de Química e Bioquímica, Faculdade de Ciências, Universidade de Lisboa, Campo Grande, 1749-016 Lisboa, Portugal; ldcortereal@fc.ul.pt (L.C.-R.); pg31015@alunos.uminho.pt (A.R.B.); adpilon@fc.ul.pt (A.P.); mhgarcia@fc.ul.pt (M.H.G.); 2 Centre of Molecular and Environmental Biology, Department of Biology, University of Minho, Campus de Gualtar, 4710-057 Braga, Portugal; apreto@bio.uminho.pt; 3 Institute of Science and Innovation for Bio-Sustainability, University of Minho, Campus de Gualtar, Edifício 18, 4710-057 Braga, Portugal; 4 i3S—Instituto de Investigação e Inovação em Saúde, Universidade do Porto, 4200-135 Porto, Portugal; nmendes@ipatimup.pt (N.M.); aribeiro@ipatimup.pt (A.S.R.); fgartner@ipatimup.pt (F.G.); 5 Centro de Química Estrutural, Institute of Molecular Sciences and Department of Chemical Engineering, Instituto Superior Técnico, Universidade de Lisboa, Av. Rovisco Pais, 1049-001 Lisboa, Portugal; tiagodmartins@tecnico.ulisboa.pt (T.D.M.); farinha@tecnico.ulisboa.pt (J.P.S.F.); conceicao.oliveira@tecnico.ulisboa.pt (M.C.O.)

**Keywords:** ruthenium-cyclopentadienyl compounds, triple negative breast cancer, active-targeting

## Abstract

The need for new therapeutic approaches for triple-negative breast cancer is a clinically relevant problem that needs to be solved. Using a multi-targeting approach to enhance cancer cell uptake, we synthesized a new family of ruthenium(II) organometallic complexes envisaging simultaneous active and passive targeting, using biotin and polylactide (PLA), respectively. All compounds with the general formula, [Ru(η^5^-CpR)(P)(2,2′-bipy-4,4′-PLA-biotin)][CF_3_SO_3_], where R is -H or -CH_3_ and P is P(C_6_H_5_)_3_, P(C_6_H_4_F)_3_ or P(C_6_H_4_OCH_3_)_3_, were tested against triple-negative breast cancer cells MDA-MB-231 showing IC_50_ values between 2.3–14.6 µM, much better than cisplatin, a classical chemotherapeutic drug, in the same experimental conditions. We selected compound **1** (where R is H and P is P(C_6_H_5_)_3_), for further studies as it was the one showing the best biological effect. In a competitive assay with biotin, we showed that cell uptake via SMVT receptors seems to be the main transport route into the cells for this compound, validating the strategy of including biotin in the design of the compound. The effects of the compound on the hallmarks of cancer show that the compound leads to apoptosis, interferes with proliferation by affecting the formation of cell colonies in a dose-dependent manner and disrupts the cell cytoskeleton. Preliminary in vivo assays in N: NIH(S)II-nu/nu mice show that the concentrations of compound **1** used in this experiment (maximum 4 mg/kg) are safe to use in vivo, although some signs of liver toxicity are already found. In addition, the new compound shows a tendency to control tumor growth, although not significantly. In sum, we showed that compound **1** shows promising anti-cancer effects, bringing a new avenue for triple-negative breast cancer therapy.

## 1. Introduction

Cancer is the second-leading cause of death worldwide, accounting for more than 10 million deaths in 2020 (World Health Organization data). Breast cancer is among the most common diseases, whereas triple negative breast cancer still lacks effective treatments.

One of the key challenges in the treatment of cancer lies in engineering drug delivery systems capable of specifically and efficiently targeting the diseased cells without affecting healthy cells/tissues [1,2,3,4]. This might be achievable using a multi-targeting approach that is able to enhance the presence of the drug in tumor tissues with target-specific and localized action. Over the past several decades, remarkable progress has been made in the development of delivery systems to treat cancer more effectively [5]. Of these systems, polymer–drug conjugates are emerging as an important class of anti-cancer drugs [6,7,8,9,10]. Indeed, the use of polymer–drug conjugates has already proven to be a successful strategy to overcome some limitations of the free drug [11]. For example, polymer–drug conjugates can improve drug uptake by cancer cells and tumors due to an enhanced permeation and retention (EPR) effect [12] while the addition of tumor-targeting vectors allows ligand-mediated targeting through specific ligand–receptor interactions (active targeting) maximizing the drug-loading into the cancer cells/tumors [13].

There are several physical encapsulation strategies using polymer micelles [14,15,16,17,18], nanoparticles [19,20,21,22] or polymer-decorated liposomes and lipid hybrids [23,24,25,26,27]. In these approaches, a fine balance between stability and drug release must be achieved in order to release the drug at its site of action. An alternative strategy consists of the covalent conjugation of the drug to the polymer. In this frame, several examples have emerged in the last years [28,29,30,31,32]. In particular, since ruthenium compounds have consistently presented lower systemic toxicity, inherent selectivity for cancer and different mechanisms of action (that can involve multiple targets) than the traditional platinum-based drugs, several polymer–ruthenium conjugates have been developed. For example, the NAMI-A copolymer conjugate based on poly(4-vinyl imidazole) and poly(ethylene glycol)methyl ether acrylate increased the cytotoxicity and cell uptake of this drug [14]. The cytotoxicity of the micelles was tested on ovarian (A2780 and Ovcar-3) and pancreatic (AsPC-1) cancer cell lines and, on average, a 1.5-fold increase in cytotoxicity was found for NAMI-A copolymer micelles when compared to NAMI-A alone. Furthermore, the NAMI-A micelles were shown to have an improved in vitro antimetastatic potential in comparison to free NAMI-A [14].

Another example of a ruthenium-metal conjugate is a drug carrier for RAPTA-C, developed by Stenzel et al., based on poly(2-hydroxyethyl acrylate) (PHEA) and poly(D,L-lactic acid) (PLA) capable of self-assembling into polymeric micelles encapsulating the ruthenium drug [33]. In terms of cytotoxicity, RAPTA-C based micelles were 10-fold more cytotoxic than the free RAPTA-C molecule in three ovarian cancer cell lines (A2780, A2780CisR and Ovcar-3) [33]. Furthermore, a significant increase in the cell uptake of ruthenium was found for the micelles compared to RAPTA-C. The same group has later reported the use of *D*-fructose micelles [34]. They observed that the micelles lead to an increased uptake by breast cancer cells (MCF-7 and MDA-MB-231) in comparison to mouse macrophages (RAW264.7) and that this uptake was higher for the glycol-based micelles than for the glycol-free micelles. These differences were attributed to the presence of GLUT5 transporters that would facilitate the uptake of the glycol-coated micelles. The authors also showed that the fructose-coated micelles presented improved antimetastatic activity.

In addition, in the frame of photodynamic therapy (a therapy that involves a photosensitizer and a light source to kill abnormal cells), the conjugation of the Ru-cytotoxic drug to a polymer has been proven to be advantageous [35,36,37,38]. In particular, Chao and Gilles and co-workers reported the encapsulation of a Ru(II) polypyridyl complex ([Ru(2,2′-bipyridine)_2_((*E*,*E′*)-4,4′-bis[*p*-methoxystyryl]-2,2′-bipyridine)]^2+^) into polymeric nanoparticles with terminal biotin groups (1,2-distearoyl-*sn*-glycero-3-phosphoethanolamine-N-[biotin(poly(ethyleneglycol))-2000][ammonium salt] (DSPE-PEG2000-biotin)) [38]. The particles showed much higher selectivity for cancer cells in comparison to noncancerous cells in 2D and 3D tumor models. Upon intravenous injection, an improved accumulation (up to 8.7) of the particles vs. the Ru complex inside an adenocarcinoma human alveolar basal epithelial tumor (A549) of a mouse was observed.

**Figure 1:**
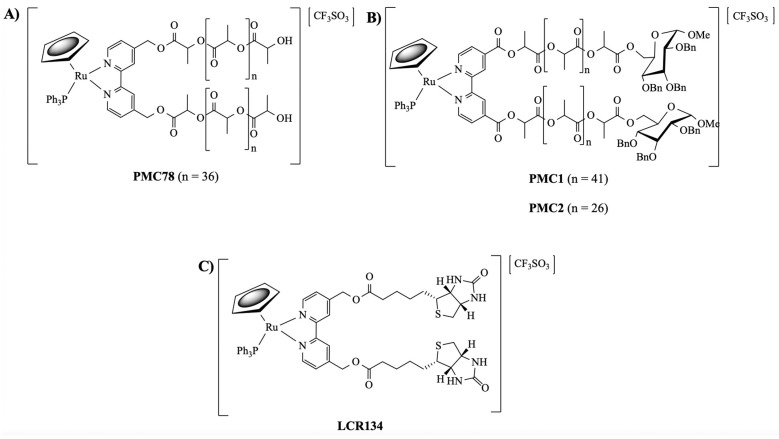
Structures of second-generation RuPMC’s. (**A**): Passive targeting effect—PMC78; (**B**): Dual targeting effect—PMC1 and PMC2 and (**C**): Active targeting effect—**LCR134**.

Our research group has been actively engaged in the design of second-generation compounds that adopt active and/or passive targeting strategies. It started with the development of a new family of polymer ‘RuCp’ conjugates (RuPMC’s) as potential anti-cancer agents [39,40]. These complexes were developed based on promising in vitro results obtained for their low molecular weight parent compound TM34, [Ru(η^5^-C_5_H_5_)(bipy)(PPh_3_)][CF_3_SO_3_] [41,42]. The rationale behind the design of this family of compounds was to use the TM34′s very stable scaffold and incorporate polylactide, a well-established and FDA approved biodegradable and biocompatible polymer for drug delivery applications [43], to potentially benefit from the EPR effect—passive targeting [39] (Figure 1A, PMC78) or to benefit from both effects (passive and active targeting), using a *D*-glucose end-capped polylactide [39,40] (Figure 1B, PMC1 and 2).

Recently, we have reported a new family of compounds incorporating biotin (vitamin B7) into the bipyridine ligand [23,44]. From this family, **LCR134** (Figure 1C) stands out as a very promising candidate that presents a dual effect rarely found in cancer drugs, i.e., the ability to overcome multidrug resistance (through P-gp inhibition) and act as a cytotoxic agent in metastatic breast cancer cells. Importantly, we could recapitulate in vivo (zebrafish model) the compound’s ability to inhibit P-gp efflux [45].

In this work, we report the synthesis, characterization, and biological studies of the high-molecular weight analogues of the previously described family of biotinylated compounds. The rationale behind this work is to improve the already excellent properties of these complexes, particularly of **LCR134**, by the addition of a polymeric chain to benefit from both targeting strategies.

## 2. Experimental Section

All reactions and purifications were performed under nitrogen atmosphere using Schlenk techniques. All solvents were used as purchased, except for dichloromethane and *n*-hexane used for the synthetic procedures and purification that were dried using an MBRAUN solvent purification system (MB SPS-800, M Braun Inertgas-Systeme GmbH, Garching, Germany). All commercial reagents (except for D,L-lactide) were used as received and without further purification. D,L-lactide was purified by recrystallization in toluene (three times) and dried overnight in the vacuum line. NMR spectra were recorded on a Bruker Avance 400 spectrometer (Fällanden, Switzerland) at probe temperature using commercially available deuterated solvents. Chemical shifts (δ) are reported in parts per million (ppm) referenced to tetramethylsilane (δ 0.00 ppm) using the residual proton solvent peaks as internal standards. ^31^P{^1^H} NMR chemical shifts were reported downfield from external standard 85% H_3_PO_4_. The peaks multiplicity is abbreviated as follows: s (singlet), d (doublet), t (triplet), m (multiplet). Coupling constants (*J*) are reported in Hertz (Hz). All assignments were attributed using APT-^13^C{^1^H} or ^13^C{^1^H}, COSY, HMBC and HMQC NMR techniques. Infrared spectra were recorded on KBr pellets using a Mattson Satellite FT-IR spectrophotometer (Kyoto, Japan) and only relevant bands were cited in the text. Electronic spectra were obtained at room temperature on a Jasco V-660 spectrometer (Elnor, Porto, Portugal) from solutions of 10^−4^–10^−5^ M in quartz cuvettes (1 cm optical path). Electrospray Ionization High Resolution Mass Spectrometry analysis were performed at *Instituto Superior Técnico*, using a QqTOF Impact II mass spectrometer equipped with an ESI source (Bruker Daltonics, Bremen, Germany). The methanolic solutions of the complexes were acquired by direct infusion (DI) using a flow rate of 150 µL/h, and a lock mass as internal calibrant was used. The full scan mass spectra were acquired over a mass range of 300–3000 *m*/*z*, at a spectra rate of 1 Hz. Data acquisition and processing were performed using the Data Analysis 5.1 software (Bruker Daltonics).

MALDI-TOF MS analyses of polymers were performed at Unidade de Espectrometría de Masas e Proteómica (Área de Infraestruturas de Investigación—Universidade de Santiago de Compostela), using a Bruker Ultraflex III TOF/TOF mass spectrometer equipped with a Smartbeam laser. Acquisitions were performed on Lineal Positive mode. The mass spectrometer was externally calibrated using Protein1 Calib Standard. DCTB was used as the matrix for MALDI-TOF MS.

The hydrodynamic diameter of complex **1** nanoparticles was measured by DLS using a Zetasizer Nano ZS (Malvern Instruments, Brookhaven, NY, USA). The nanoparticle aqueous dispersions were diluted as required and measured at room temperature.

### 2.1. Synthesis

The starting materials used in the synthesis of the organometallic complexes were prepared following the methods previously described in the literature; [RuCp(PPh_3_)_2_Cl] [46], [RuCp(P(C_6_H_4_F)_3_)_2_Cl] [23], [RuCp(P(C_6_H_4_OCH_3_)_3_)_2_Cl] [23] and [Ru(MeCp)(PPh_3_)_2_Cl] [47].

#### 2.1.1. General Procedure for the Polymerization of D,L-Lactide (Bipy-PLA-OH)

Purified D,L-lactide (1.00 g, 13.9 mmol), 4,4′-bis(hydroxymethyl)-2,2′-bipyridine (bipy-OH; 0.05 g, 0.23 mmol) and 4-dimethylaminopyridine (DMAP; 0.06 g, 0.50 mol) were added to a Schlenk vessel. The reaction vessel was placed in an oil bath at 135 °C for 15 min. with constant magnetic stirring. After that period, quenching of the reaction was performed with some drops of a water/methanol mixture (50/50 (% *v*/*v*)). Dichloromethane was then added to completely dissolve the polymer and the solution was transferred back to the water/methanol mixture. The polymeric compound was precipitated, under reduced pressure, until the solution became translucent. The solution was then decanted, and the residue was washed with diethyl ether (2 × 10 mL) and dried overnight, under vacuum, originating bipy-PLA-OH polymer (white foam) with 82-95 % yield (Table 1). ^1^H NMR [(CDCl_3_), Me_4_Si, δ/ppm]: 8.67 (d, 2, ^3^*J*_HH_ = 4.00, H_1_); 8.36 (s, 2, H_3_); 7.28 (under the signal of the solvent, H_2_); 5.16 (m, 63, H_6_+H_A_); 4.36 (m, 2, H_A’_); 1.56 (m, 189, H_B_); 1.48 (m, 6, H_B’_).

#### 2.1.2. General Procedure for the Synthesis of 2,2′-Bipyridine-4,4′-PLA-Biotin (L1)

To a stirred and degassed solution of bipy-PLA-OH (0.16 mmol) in a dichloromethane (10 mL)/DMF (3 mL) mixture, 5-[(3aS,4S,6aR)-2-oxohexahydro-1H-thieno [3,4-d] imidazole-4-yl]pentanoic acid (biotin) (0.10 g, 0.4 mmol), 4-dimethylamino-pyridine (DMAP) (0.02 g, 0.16 mmol) and N-(3-dimethylaminopropyl)-N′-ethylcarbodiimide hydrochloride (EDC) (0.08 g, 0.4 mmol) were added. The esterification reaction was followed by ^1^H-NMR spectroscopy until the terminal signal (HA’) of the PLA chain completely disappeared. After a 15 h reflux the reaction mixture was cooled down to room temperature and the solvent was removed under vacuum. The oily residue was dissolved in dichloromethane (40 mL) and extracted with MQ water (2 × 40 mL) in a separating funnel. The organic layers were collected in a round bottom flask and the solvent was evaporated under reduced pressure. The residue was washed with diethyl ether (2 × 10 mL) and dried overnight, under vacuum, originating **L1** (white foam) with 90 % yield (Table 2). ^1^H NMR [(CD_3_)_2_CO, Me_4_Si, δ/ppm]: 8.71 (d, 2, ^3^*J*_HH_ = 4, H_1_); 8.49 (s, 2, H_4_); 7.45 (d, 2, ^3^*J*_HH_ = 4.0 Hz, H_2_); 5.77 (s, 2, NH); 5.65 (s, 2, NH); 5.38 (m, 4, H_6_); 5.21 (m, 61, H_A_, PLA); 4.49 (m, 4, H_15_); 4.34 (m, 4, H_13_); 3.22 (m, 2, H_12_); 2.94 (m, 2, H_16_); 2.71 (m, 2, H_16_); 2.40 (m, 4, H_8_); 1.56 (m, 207, H_9_ + H_10_ + H_11_ + H_B_, PLA). ^13^C NMR [(CD_3_)_2_CO, δ/ppm]: 173.18 (C_7_); 170.09 (C_C_, PLA); 156.81 (C_14_); 150.43 (C_5_); 150.43 (C_1_); 146.79 (C_3_); 123.08 (C_2_); 119.75 (C_4_); 69.84 (C_A_, PLA); 65.92 (C_6_); 62.29 (C_13_); 60.71 (C_15_); 56.38 (C_12_); 41.03 (C_16_); 34.01 (C_8_); 29.84 (under the signal of the solvent, C_10_, C_11_); 25.55 (C_9_); 17.05 (C_B_, PLA). UV-vis [DMSO, λ_max_/nm (ε × 10^3^/M^−1^cm^−1^)]: 285 (11.6). FTIR [KBr, cm^−1^]: 3244 (υ_N-H_ amine), 3069 (υ_C-H_ aromatic rings); 2995-2878 (υ_C-H_ alkanes), 1757 (υ_C=O_ PLA and υ_C=O_ ester), 1705 (υ_C=O_ ketone), 1458 (υ_C=C_ aromatic rings); 1186 (υ_C-O_ ester).

#### 2.1.3. General Procedure for the Synthesis of [Ru(CpR)(P(C_6_H_4_R’)_3_)(2,2′-Bipyridine-4,4′-PLA-Biotin][CF_3_SO_3_] R = H; R’ = H (1) or R = H; R’ = F (2) or R = H; R’ = OCH_3_ (3) or R = CH_3_; R’ = H (4)

To a stirred and degassed solution of [Ru(η^5^-Cp)(PPh_3_)_2_Cl], [Ru(Cp)(P(C_6_H_4_F)_3_)_2_Cl], [Ru(Cp)(P(C_6_H_4_OCH_3_)_3_)_2_Cl] or [Ru(CpMe)(P(C_6_H_5_)_3_)_2_Cl] (0.16 g, 0.22 mmol; 0.08 g, 0.09 mmol; 0.10 g, 0.11 mmol or 0.05 g, 0.06 mmol, respectively) in dichloromethane (40 mL), **L1** (L1_PLA1: 0.57 g, L1_PLA2: 0.18 mmol; L1_PLA1: 0.35 g, 0.08 mmol; L1_PLA3: 0.30 g, 0.08 mmol and 0.23 g, 0.05 mmol for **1**, **2**, **3** and **4**, respectively) and AgCF_3_SO_3_ (0.07 g, 0.27 mmol; 0.03 g, 0.12 mmol; 0.03 g, 0.12 mmol and 0.02 g, 0.08 mmol for **1**, **2**, **3** and **4**, respectively) were added. After a 14, 19, 16 or 7 h reflux (for **1**, **2**, **3** or **4**, respectively) the reaction mixture was cooled down to room temperature, filtered and the solvent was removed under vacuum. The orange/brown residue was recrystallized several times from CH_2_Cl_2_/*n*-hexane until full confirmation of its purity by ^1^H-NMR, originating complexes **1**, **2**, **3** and **4** in 43, 63, 59 and 47 % yield, respectively (Table 3).

**Complex 1**: ^1^H NMR [(CD_3_)_2_CO, Me_4_Si, δ/ppm]: 9.52 (m, 2, H_1_); 8.09 (m, 2, H_4_); 7.45 (m, 3, H*_para_*PPh_3_); 7.34 (m, 8, H_2_+H*_meta_*PPh_3_); 7.12 (m, 6, H*_ortho_*PPh_3_); 5.35 (m, 4, H_6_); 5.20 (m, 99, H_A_, PLA); 4.95 (s, 5, η^5^-C_5_H_5_); 4.77 (m, 2, H_15_); 4.69 (m, 2, H_13_); 3.36 (m, 2, H_12_); 2.86 (m, under de signal of the water of solvent, H_16_); 2.42 (m, 4, H_8_); 1.79 (m, 4, H_9_); 1.55 (m, 179, H_10_+H_11_+H_B_, PLA). ^13^C NMR [(CD_3_)_2_CO, δ/ppm]: 173.25 (C_7_); 170.10 (C_C_, PLA); 156.94 (C_1_); 156.29 (C_5_); 146.64 (C_3_); 133.82 (d, ^2^*J*_CP_ = 11.1, CH*_ortho_*PPh_3_); 132.12 (d, ^1^*J*_CP_ = 42.3, Cq, PPh_3_); 131.09 (CH*_para_*PPh_3_); 129.42 (d, ^3^*J*_CP_ = 10.1, CH*_meta_*PPh_3_); 123.95 (C_2_); 122.04 (C_4_); 79.42 (Cp); 69.82 (C_A_, PLA); 65.02 (C_6_); 62.55 (C_13_); 60.58 (C_15_); 58.14 (C_12_); 42.65 (C_16_); 33.90 (C_8_); 29.84 (under the signal of the solvent, C_10_, C_11_); 25.32 (C_9_); 17.06 (C_B_, PLA). ^31^P NMR [(CD_3_)_2_CO, δ/ppm]: 51.13 (s, PPh_3_). UV-vis [CH_2_Cl_2_, λ_max_/nm (ε × 10^3^/M^−1^cm^−1^)]: 295 (29.5), 336 (8.1), 430 (4.8), 481 (Sh). [DMSO, λ_max_/nm (ε × 10^3^/M^−1^cm^−1^)]: 296 (35.5), 361 (Sh), 426 (5.7), 477 (Sh). FTIR [KBr, cm^−1^]: 3416 (υ_N-H_ amine), 3075 (υ_C-H_ Cp and aromatic rings); 2995-2878 (υ_C-H_ alkanes), 1757 (υ_C=O_ PLA and υ_C=O_ ester), 1454 (υ_C=C_ Cp and aromatic rings), 1273, 1186, 1030 (υ(CF_3_SO_3_^−^)), 1186 (υ_C-O_ ester). Mn, calculated by NMR: 6233.7 g/mol.
**Complex 2**: ^1^H NMR [(CD_3_)_2_CO, Me_4_Si, δ/ppm]: 9.54 (m, 2, H_1_); 8.21 (m, 2, H_4_); 7.41 (m, 2, H_2_); 7.15 (m, 12, P(PhF)_3_); 5.36 (m, 4, H_6_); 5.20 (m, 85, H_A_, PLA); 5.00 (s, 5, η^5^-C_5_H_5_); 4.69 (m, 2, H_15_); 4.61 (m, 2, H_13_); 3.24 (m, 2, H_12_); 2.85 (m, under de signal of the water of solvent, H_16_); 2.41 (m, 4, H_8_); 1.70 (m, 4, H_9_); 1.55 (m, 278, H_10_+H_11_+H_B_, PLA). ^13^C NMR [(CD_3_)_2_CO, δ/ppm]: 173.11 (C_7_); 170.10 (C_C_, PLA); 164.76 (dd, ^1^*J*_CF_ = 250.5; Cq, P(PhF)_3_); 157.14 (C_1_); 156.32 (C_5_); 147.03 (C_3_); 136.22 (dd, ^3^*J*_CP_ = 12.6; ^2^*J*_CF_ = 9.0, CH*_meta_*P(PhF)_3_); 128.20 (dd, ^1^*J*_CP_ = 42.3, Cq, P(PhF)_3_); 124.35 (C_2_); 122.42 (C_4_); 116.64 (dd, ^2^*J*_CP_ = 21.1; ^3^*J*_CF_ = 11.1 Hz, CH*_ortho_*P(PhF)_3_); 79.66 (Cp); 69.82 (C_A_, PLA); 65.07 (C_6_); 62.60 (C_13_); 60.57 (C_15_); 58.21 (d, C_12_); 43.11 (C_16_); 33.91 (C_8_); 29.84 (under the signal of the solvent, C_10_, C_11_); 25.33 (d, C_9_); 17.06 (C_B_, PLA). ^31^P NMR [(CD_3_)_2_CO, δ/ppm]: 50.09 (s, P(PhF)_3_. UV-Vis [CH_2_Cl_2_, λ_max_/nm (ε × 10^3^/M^−1^cm^−1^)]: 295 (30.2); 336 (7.3); 427 (4.9); 479 (Sh). [DMSO, λ_max_/nm (ε × 10^3^/M^−1^cm^−1^)]: 296 (27.0); 346 (Sh); 426 (4.2); 478 (Sh). FTIR [KBr, cm^−1^]: 3420 (υ_N-H_ amine); 3069 (υ_C-H_ Cp and aromatic rings); 2997–2876 (υ_C-H_ alkanes), 1757 (υ_C=O_ PLA and υ_C=O_ ester), 1454 (υ_C=C_ Cp and aromatic rings), 1284, 1186, 1028 (υ(CF_3_SO_3_^−^)), 1186 (υ_C-O_ ester). Mn, calculated by NMR: 5941.6 g/mol.
**Complex 3**: ^1^H NMR [(CD_3_)_2_CO, Me_4_Si, δ/ppm]: 9.52 (d, 2, ^3^*J*_HH_ = 8.0, H_1_); 8.14 (s, 2, H_4_); 7.37 (m, 2, H_2_); 7.02 (m, 6, H*_ortho_*P(PhOCH_3_)_3_); 6.87 (m, 6, H*_meta_*P(PhOCH_3_)_3_); 5.35 (m, 4, H_6_); 5.22 (m, 86, H_A_, PLA); 4.93 (s, 5, η^5^-C_5_H_5_); 4.52 (m, 2, H_15_); 4.36 (m, 2, H_13_); 3.83 (s, 9, OCH_3_); 3.26 (m, 2, H_12_); 2.85 (m, under the signal of the water of solvent, H_16_) 2.40 (m, 4, H_8_), 1.55 (m, 278, H_9_+H_10_+H_11_+H_B_, PLA). ^13^C NMR [(CD_3_)_2_CO, δ/ppm]: 173.20 (C_7_); 170.10 (C_C_, PLA); 161.78 (Cq, P(PhOCH_3_)_3_); 156.91 (C_1_); 156.31 (C_5_); 146.12 (C_3_); 135.29 (d, ^2^*J*_CP_ = 13.1, CH*_ortho_*P(PhOCH_3_)_3_); 123.87 (C_2_); 123.52 (d, ^1^*J*_CP_ = 47.3, Cq, P(PhOCH_3_)_3_); 122.36 (C_4_); 114.76 (d, ^3^*J*_CP_ = 10.1, CH*_meta_*P(PhOCH_3_)_3_); 79.20 (Cp); 69.82 (C_A_, PLA); 65.08 (C_6_); 62.36 (C_13_); 60.73 (C_15_); 58.01 (C_12_); 55.70 (OCH_3_); 39.75 (C_16_); 33.97 (C_8_); 29.84 (under the signal of the solvent, C_10_, C_11_); 25.49 (C_9_); 17.05 (C_B_, PLA). ^31^P NMR [(CD_3_)_2_CO, δ/ppm]: 47.02 (s, P(PhOCH_3_)_3_). UV-vis [CH_2_Cl_2_, λ_max_/nm (ε × 10^3^/M^−1^cm^−1^)]: 246 (53.8); 295 (20.2); 336 (5.9); 435 (3.2); 492 (Sh). [DMSO, λ_max_/nm (ε × 10^3^/M^−1^cm^−1^)]: 294 (18.9); 341 (Sh); 433 (2.9); 492 (Sh). FTIR [KBr, cm^−1^]: 3410 (υ_N-H_ amine); 3073 (υ_C-H_ Cp and aromatic rings); 2995–2878 (υ_C-H_ alkanes); 1757 (υ_C=O_ PLA and υ_C=O_ ester), 1456 (υ_C=C_ Cp and aromatic rings), 1278, 1186, 1028 (υ(CF_3_SO_3_^−^)), 1186 (υ_C-O_ ester). Mn, calculated by NMR: 4319.4 g/mol.
**Complex 4**: ^1^H NMR [(CD_3_)_2_CO, Me_4_Si, δ/ppm]: 9.47 (m, 2, H_1_); 8.11 (m, 2, H_4_); 7.42 (m, 4, H_2_+H*_para_*PPh_3_); 7.33 (m, 7, H*_meta_*PPh_3_); 7.11 (m, 6, H*_ortho_*PPh_3_); 5.36 (m, 4, H_6_); 5.20 (m, 64, H_A_, PLA); 4.77 (s, 2, H_d_, Cp); 4.71 (m, 2, H_15_); 4.67 (m, 2, H_c_, Cp); 4.55 (m, 2, H_13_); 3.39 (m, 2, H_12_); 3.05 (m, 2, H_16_); 2.88 (m, 2, H_16_); 2.40 (m, 4, H_8_); 1.68 (m, 7, H_9_ + H_a_, Cp); 1.55 (m, 211, H_10_+H_11_+H_B_, PLA). ^13^C NMR [(CD_3_)_2_CO, δ/ppm]: 173.14 (C_7_); 170.09 (C_C_, PLA); 156.49 (C_1_); 156.22 (C_5_); 146.44 (C_3_); 133.75 (d, ^2^*J*_CP_ = 11.1, CH*_ortho_*PPh_3_); 132.29 (d, ^1^*J*_CP_ = 41.3, Cq, PPh_3_); 131.01 (CH*_para_*PPh_3_); 129.40 (d, ^3^*J*^CP^ = 10.1, CH*_meta_*PPh_3_); 124.32 (C_2_); 123.72 (C_4_); 103.38 (C_b_, Cp); 77.00 (C_c_ + C_d_, Cp); 69.81 (C_A_, PLA); 64.99 (C_6_); 63.27 (C_13_); 61.74 (C_15_); 56.47 (C_12_); 40.89 (C_16_); 33.95 (C_8_); 29.84 (under the signal of the solvent, C_10_, C_11_); 25.41 (C_9_); 17.05 (C_B_, PLA); 11.69 (C_a_, Cp). ^31^P NMR [(CD_3_)_2_CO, δ/ppm]: 51.63 (s, PPh_3_). UV-vis [CH_2_Cl_2_, λ_max_/nm (ε × 10^3^/M^−1^cm^−1^)]: 294 (27.1), 334 (9.0), 435 (4.8), 489 (Sh). [DMSO, λ_max_/nm (ε × 10^3^/M^−1^cm^−1^)]: 296 (12.6), 343 (3.8), 431 (2.1), 493 (Sh). FTIR [KBr, cm^−1^]: 3307 (υ_N-H_ amine), 3069 (υ_C-H_ Cp and aromatic rings); 2997–2878 (υ_C-H_ alkanes); 2878 (υ_C-H_ Cp and aromatic rings); 1753 (υ_C=O_ PLA and υ_C=O_ ester), 1456 (υ_C=C_ Cp and aromatic rings), 1277, 1186, 1028 (υ(CF_3_SO_3_^-^)), 1186 (υ_C-O_ ester). Mn, calculated by NMR: 5296.0 g/mol.

### 2.2. Biological Studies

#### 2.2.1. Cell Lines and Culture Conditions

MDA-MB-231 cells were grown at 37 °C in 5% CO_2_ in Dulbecco’s modified Eagle’s medium (DMEM high glucose) (Capricorn Scientific, Ebsdorfergrund, Germany) supplemented with 10% fetal bovine serum (Capricorn Scientific) and 1% penicillin/streptomycin (Capricorn Scientific).

#### 2.2.2. Compounds Dilution and Storage

All compounds were dissolved in 30% DMSO/70% MQ H_2_O and filtered (40 μm filters), followed by a serial dilution to the corresponding tested concentrations of the Ru-PLA conjugates (compounds **1**–**4**). The stock solutions of the compounds were divided in aliquots of 10 μL each and stored at −80 °C until further use.

#### 2.2.3. MTT Assay

The cells were adherent in monolayers and, upon confluency, were harvested by digestion with trypsin-EDTA. The cytotoxicity of the complexes against MDA-MB-231 was assessed using the colorimetric assay MTT (3-(4,5-2-yl)-2,5-ditetrazolium bromide), which measures the conversion of the yellow tetrazolium into purple formazan by mitochondrial redox activity in living cells. For this purpose, cells (10–20 × 10^3^ in 200 µL of medium) were seeded into 96-well plates and incubated in a 5% CO_2_ incubator at 37 °C. Cells settled for 24 h followed by the addition of a dilution series of the complexes in medium (200 µL). The complexes and ligand were first solubilized in 100% DMSO, given a 10 mM stock solution, then in medium within the concentration range 0.01–50 µM for the complexes and 10–200 µM for the ligand. DMSO did not exceed 1% even for the higher concentration used and was without cytotoxic effect. After 48 h incubation, the treatment solutions were removed by aspiration and MTT solution (200 µL, 0.5 mg/mL in PBS) was added to each well. After 3–4 h at 37 °C/5% CO_2_, the solution was removed, and the purple formazan crystals formed inside the cells were dissolved in DMSO (200 µL) by thorough shaking. The cellular viability was evaluated by measuring the absorbance at 570 nm by using a microplate spectrophotometer.

#### 2.2.4. Intracellular Distribution

For the cellular uptake experiments, MDA-MB-231 cells (*ca.* 1 × 10^6^ in 5 mL medium) were seeded into t25 flasks and incubated in a 5% CO_2_ incubator at 37 °C. Cells settled for 24 h, followed by the addition of complex 1 at a concentration equivalent to its IC_50_ value found for 48 h challenge at 37 °C, with or without a pre-incubation with free biotin (1 mg/mL) for 1 h. After complex incubation for 3 h, cells were washed with ice-cold PBS and treated in order to obtain a cellular pellet. The cytosol, membrane/particulate, cytoskeletal and nuclear fractions were extracted using a FractionPREP^TM^ (BioVision, Milpitas, CA, USA) cell fractionation kit according to the manufacturer’s protocol. The Ru (^101^Ru) content in each fraction was measured by a Thermo X-Series Quadrupole ICPMS (Thermo Scientific, Waltham, MA, USA) after digestion of the samples and using the same procedure previously described [49].

#### 2.2.5. Colony Formation Assay

MDA-MB-231 cells were seeded at a concentration of 300 cells/mL, in six-well plates. After 24 h of seeding, cells were incubated with different concentrations of compound **1** and cisplatin for 48 h. The negative control cells were treated with DMSO (maximum of 0.1% of DMSO per well (*v*/*v*)) and H_2_O (maximum of 0.3% H_2_O per well (*v*/*v*)). After 48 h of incubation, the medium was replaced by fresh medium without compound **1**. The medium was renewed every 3 days. Eleven days after removing the treatments, cells were stained as previously described [23].

#### 2.2.6. F-Actin Immunofluorescence Assay

MDA-MB-231 cell line was seeded at a concentration of 4.2 × 10^4^ cells/mL, in 12-well plates with one coverslip per well. After 24 h, cells were exposed to the IC_50_ values of compound **1** and cisplatin. The negative control cells were treated with DMSO (maximum of 0.1% of DMSO per well (*v*/*v*)) and H_2_O (maximum of 0.3% H_2_O per well (*v*/*v*)). After 48 h of incubation, the cells were washed twice with PBS and fixed with paraformaldehyde 4% (*w*/*v*) for 10 min. After fixation, cells were incubated with NH_4_Cl 50 mM and washed twice with PBS, for five minutes. The cells were permeabilized using Triton X-100 0.2%, for five minutes, and blocked with PBS–BSA 3% for 20 min. Subsequently, cells were incubated with Alexa Fluor^TM^ 488 Phalloidin (ThermoFisher Scientific^®^, Waltham, MA, USA), diluted in PBS (1:40), for one hour in the dark, and washed twice with PBS. To finalize, coverslips were mounted using 5 μL of VECTASHIELD Antifade Mounting Medium with DAPI (Vector Laboratories^®^, Peterborough, UK) in microscope slides. Representative images were obtained in a fluorescence microscope (Olympus motorized BX63F Upright Microscope) (Olympus©, Tokyo, Japan) at a magnification of 600×.

#### 2.2.7. Evaluation of the Cell Death Mechanism by Flow Cytometry

The triple-negative breast cancer cell line MDA-MB-231 was seeded in 6-well test plates at a concentration of 4.2 × 10^4^ cells/mL. 24 h later, cells were incubated with different concentrations of compound **1** and cisplatin for 48 h. After the incubation period, cells were collected and stained as previously described [23] Samples were analyzed using FlowJo 7.6 software.

#### 2.2.8. Statistical Analysis for In Vitro Studies

The results were obtained from at least three independent experiments and expressed as mean ± SD. For analyzing the results were used a one-way or two-way ANOVA with Dunnett’s post-test. *p*-values lower than 0.05 were considered statistically significant. All statistical analyses were performed using GraphPad Prism version 8 for macOS, GraphPad Software, San Diego, CA, USA, www.graphpad.com.

### 2.3. In Vivo Studies

For this assay, the nude mice strain produced, housed and bred at i3S animal House (N: NIH(S)II-nu/nu mice) was maintained in a pathogen-free environment, under controlled conditions of light and humidity. The current experimental set-up was conducted with the application of the 3Rs (replacement, reduction, and refinement) and determined group of animals was established to use the minimal number of animals needed for a correct statistical analysis of the data. This project has been previously approved by the animal ethics committee and animal welfare body of i3S (Project FG-2016-01.

For the toxicity assay, the mice (3 per group) were treated, twice a week, by intraperitoneal (IP) injection with a mixture of 30% DMSO and 20% of an aqueous solution of Captisol^®^ for complex **LCR134** and a mixture of 30% DMSO/70% MQ water for complex **1** (vortexed before each administration) at 4 different drug dosages (0.5 mg/kg, 1.5 mg/kg, 3 mg/kg and 4 mg/kg). Two weeks after, blood was collected from all mice groups by intracardiac puncture and after cervical dislocation, a necropsy was performed on all mice in order to collect target organs for further histological analysis to search for signs of cytotoxic effects.

For the spontaneous tumor growth, orthotopic injection into the mammary fat pad of female mice, with 6–8 weeks of age, with 5 × 10^6^ MDA-MB-231 cells re-suspended in DMEM cell culture media, using a 25 G needle was performed. Mice were weighed and tumor width and length were measured with calipers, twice a week. Tumor volume was estimated by using the equation, V = 0.5 × a × b^2^, where V is the volume, a is the length of the major axis of the tumor, and b is the length of its minor axis. Whenever the tumors reached a mean volume of 500–1000 mm^3^, they were surgically removed, fixed in 10% buffered formalin and then embedded in paraffin, sectioned, and stained with hematoxylin and eosin.

At the end of the experiment, mice were euthanized, and a necropsy was performed to collect target metastatic organs (lymph nodes, lung and liver). All the collected organs were immediately fixed in 10% buffered formalin, histologically processed and embedded in paraffin, sectioned, and stained with hematoxylin and eosin (H&E) for histopathological evaluation.

#### Statistical Analysis

Statistical analysis was carried out using SPSS statistics V.17.0 software package for Windows (SPSS, Inc., Chicago, IL, USA) and Graph Pad Prism version 9.0c software (Graph Pad Software, San Diego, CA, USA). P-values lower than 0.05 will be considered statistically significant. All statistical tests will be two-sided.

## 3. Results and Discussion

### 3.1. Synthesis and Characterization of Ruthenium Compounds

**Scheme 1:**
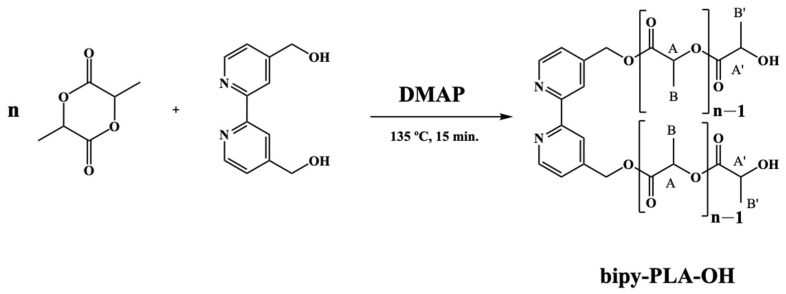
Ring-opening polymerization (ROP) of D,L-lactide, using bipy-OH as initiator and DMAP as catalyst, originating ligand bipy-PLA-OH.

**Scheme 2:**
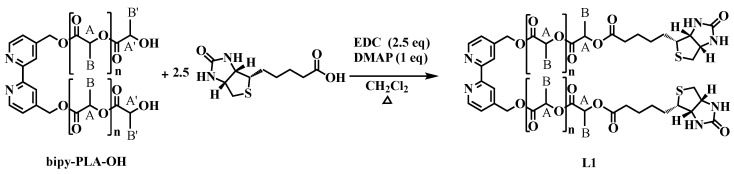
Esterification reaction between bipy-PLA-OH and biotin, using EDC and DMAP as coupling agent and catalyst, respectively, originating ligand **L1**.

Given the successful results previously obtained for a series of biotinylated ruthenium−cyclopentadienyl complexes [23,44,45] aimed for the active targeting of cancer cells, a new family of ruthenium(II) organometallic complexes was designed envisaging simultaneous active and passive targeting, using biotin and polylactide (PLA), respectively. All compounds have the general formula, [Ru(η^5^-CpR)(P)(2,2′-bipy-4,4′-PLA-biotin)][CF_3_SO_3_], where R is -H or -CH_3_ and P is P(C_6_H_5_)_3_, P(C_6_H_4_F)_3_ or P(C_6_H_4_OCH_3_)_3_, and were successfully synthesized and isolated as triflate salts. The ligand 2,2′-bipyridine-4,4′-PLA-biotin (bipy-PLA-biotin), **L1**, was obtained in two steps. First, the macromolecular ligand 2,2′-bipyridine-4,4′-PLA-OH (bipy-PLA-OH) was obtained by ring-opening polymerization (ROP) of D,L-lactide (rac-lactide) in bulk, using 4,4′-bis(hydroxymethyl)-2,2′-bipyridine (bipy-OH) as initiator and 4-dimethylaminopyridine (DMAP) as catalyst, at 135 °C for 15 min (Scheme 1) [39]. The size of the polymer can be controlled by the monomer:initiator (bipy-OH) ratio. Ligand **L1** was later obtained by an esterification reaction between bipy-PLA-OH and biotin, using N-(3-dimethylaminopropyl)-N′-ethylcarbodiimide hydrochloride (EDC) and DMAP as coupling agent and catalyst, respectively, as shown in Scheme 2, in high yield (90%).

**Scheme 3:**
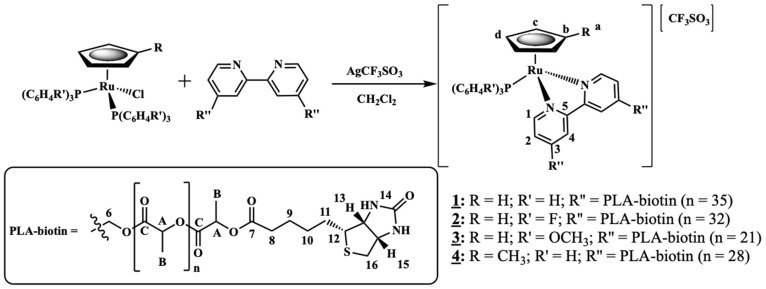
Synthetic route of the new Ru(II)-PMC-biotin complexes **1**–**4**. Compounds are numbered for NMR assignment.

The complexes were obtained by σ coordination of the bidentate ligand, **L1** to ruthenium. The compounds were achieved in moderate to good yields (43–63%) by halide abstraction from the precursors (Scheme 3).

Analysis of the overall ^1^H NMR data show that the resonances of the Cp ring appear as one signal corresponding to the equivalent protons of the non-substituted η^5^-Cp ligand for complexes **1**–**3** (δ 4.93–5.00 ppm) or as two singlets (δ 4.67 and 4.77 ppm) for complex **4**, due to the two nonequivalent protons of the η^5^-MeCp ligand. A deshielding effect on the Cp upon coordination of **L1** ligand was observed, as expected for monocationic ruthenium(II) complexes. An evident deshielding on the H_1_ protons (δ 0.76–0.83 ppm) and a shielding on the H_4_ protons (δ 0.28–0.40 ppm) for all complexes is also observed, clear evidence of successful coordination of **L1**. The downfield shift of H_1_ protons is slightly less pronounced (δ 0.76 vs. ~0.81 ppm) for compound **4** due to the influence of the donating methyl group on the Cp ring. The shielding effect on the H_4_ protons is due to the π-backdonation of the organometallic fragment to the bipyridyl ligand and is slightly less shielded (δ 0.28 vs. ~0.40 ppm) for compound **2** due to the withdrawing character of the fluorine at *para* position of the phosphane ligand. The ^31^P NMR spectra showed a unique sharp singlet for all the compounds at ~50 ppm. Complex **3** containing 4-(methoxyphenyl)phosphane has the most shielded signal (δ 47.02 ppm), due to the presence of the strong electron donating group, -OCH_3_ at the *para* position of the benzene ring.

The APT-^13^C{1H} and ^13^C{_1_H} NMR data are in accordance with the aforementioned effects in the ^1^H NMR analysis.

Experimental average molecular weights were calculated by NMR end group analysis (integral of the ^1^H NMR peak of proton A vs. integral of the ^1^H NMR peak of proton 1) [48]. The MALDI-TOF analysis reveals a distribution of peaks, spaced by the expected Dm/z = 72.02 (Figures S18–S21). Besides this, the isotopic pattern of each peak clearly shows the presence of ruthenium. As reported elsewhere, these polymers cannot be analyzed by size exclusion chromatography (SEC) with refractive index detector (RID), possibly by the interaction of the metal ions and nitrogen atoms with the SEC column material [37,50]. As such, to complete the polymer-metal conjugates characterization, methanolic solutions of the complexes were also analyzed by QTOFMS with an ESI ion source. The high-resolution mass spectra displayed series of double charged ions exhibiting the characteristic *m/z* 72.02 spacing of the catalyst-bound PLA, the measured isotopic patterns in agreement with those calculated for the proposed polymer–metal conjugates (Figures S22–S25).

**Figure 2:**
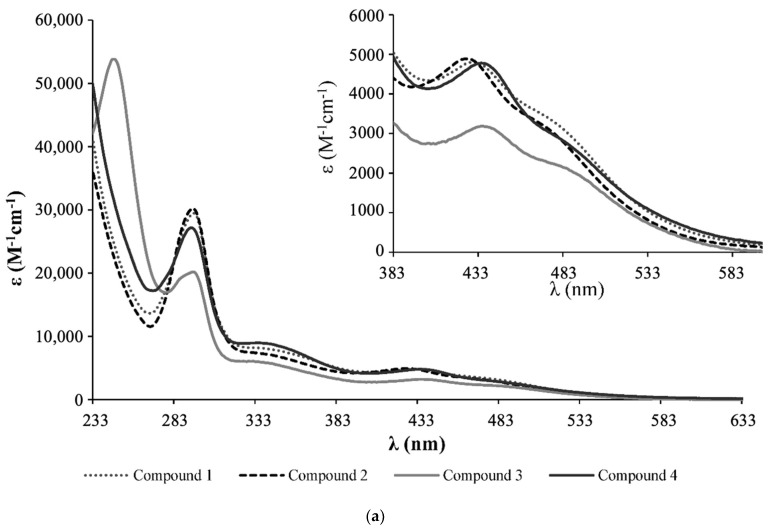

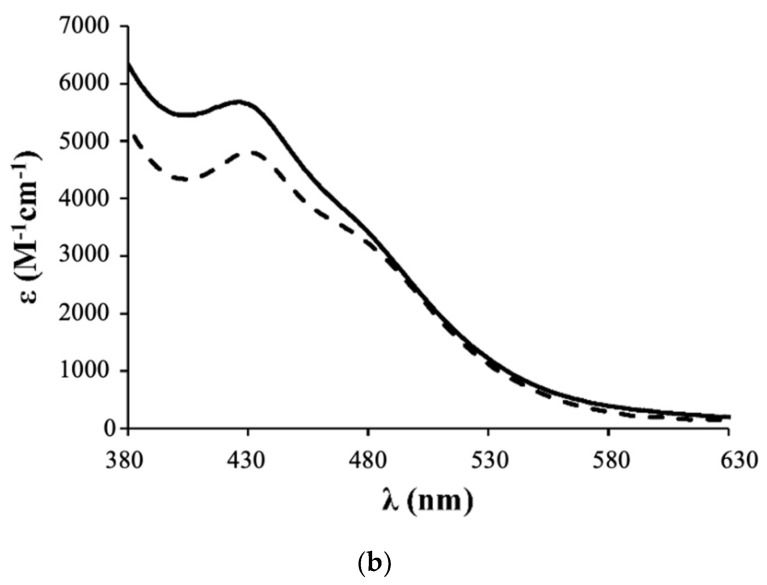
Electronic spectra of complexes (**a**) **1** (dotted line), **2** (dashed line), **3** (grey solid line) and **4** (black solid line) in dichloromethane solutions. Expansion of the spectra in the region of the CT transitions (**b**) of complex **1** in DMSO (solid line) and in dichloromethane (dashed line) solutions, showing the hypsochromic shift.

The optical absorption spectra of complexes **1**–**4** were recorded in 10^−4^ to 10^−5^ M in dichloromethane and dimethyl sulfoxide solutions. All compounds present an intense UV band below 250 nm attributed to π→π* transitions occurring at the organometallic fragment {RuCp(phosphane)}^+^, with a maximum wavelength at ~246 nm for complex **3**, containing 4-(methoxyphenyl)phosphane (Figure 2a); this band is below the cut-off of the solvent for the remaining compounds. A second strong absorption band in the UV region is observed, appearing at λ ~295 nm and can be attributed to the π→π* transitions that take place in the coordinated bipy-PLA-biotin ligand. There is also a low-intensity band for the same complexes, appearing at λ = ~336 nm, that may be attributed to a π(Ru + Cp) →π*(bipyridyl+phosphane) transition. In addition to these bands, two maximum absorption bands (427–435 nm and 479–492 nm) were found. Due to their intensity and position, they may be attributed to metal-to-ligand charge transfer bands (MLCT), from Ru 4d orbitals to π* orbitals of N-heteroaromatic rings and to phosphane, as previously reported for related compounds [51]. Electronic spectra in DMSO were performed in order to infer about the charge transfer character of these bands. A hypsochromic shift of ca. 4 nm at the MLCT band can be observed, confirming the charge transfer nature of it, as exemplified in Figure 2b for compound **1**.

The solid-state FT-IR spectra (KBr pellets) of **L1** and complexes **1**–**4** show the presence of the typical bands attributed to υ_N-H_ stretching of amines in the range 3244–3420 cm^−1^ and the bands for the υ_C-H_ aromatic and υ_C-H_ alkanes stretching in the range 3069–3075 cm^−1^ and 2995–2878 cm^−1^, respectively. The υ_C=O_ stretching of the ester and ketone at ~1757 cm^−1^ was also present as well as the typical bands attributed to the triflate counterion at ~1273, 1186 and 1029 cm^−1^.

### 3.2. Biological Evaluation of the Compounds

#### 3.2.1. Analysis of the Anti-Cancer Effect in MDA-MB-231 Breast Cancer Cells

The effect on cell viability of compounds **1**−**4** and of **L1** was assessed in MDA-MB-231 breast cancer cell line, using the colorimetric MTT assay. This cell line was chosen since it displays many phenotypic/genotypic resemblances with the triple-negative breast cancer model (TNBC), such as the lack of expression of estrogen receptor (ER), progesterone receptor (PR) and the absence of human epidermal growth factor Receptor-2 (HER2). This high-incidence and prevalent type of cancer has no currently available treatments and the mortality rates are very high, highlighting the urgent need to find novel therapeutic strategies.

Cells were treated with the compounds within the concentration range of 0.01−50 μM (or 10−200 μM for **L1**), for a period of 48 h (Table 4). The IC_50_ values of the non-polymeric analogues (determined using the same method) [Ru(η^5^-CpR)(P(C_6_H_4_R’)_3_)(bipy-biotin)]^+^ (**LCR134**: R = Cp and R’ = H; **LCR222**: R = Cp and R’ = F; **LCR239**: R = Cp and R’ = OCH_3_) [23] and CDDP were also included for comparison. We can observe that all the organometallic compounds display very low IC_50_ values (<15 μM), being much more potent than the organic ligands (**L1** and phosphanes [23]). Furthermore, all compounds exhibit lower IC_50_ values than CDDP by ~5−31 fold. Interestingly, compound **1** is the most potent, following the same trend as its low-molecular weight analogue (**LCR134**). Overall, these results show that the introduction of the PLA ligand as well as the synergy between all co-ligands have an important influence on the compounds’ final anticancer effect, suggesting that these compounds might be potent anti-cancer drugs as the dose that shows inhibition of cell viability is low.

#### 3.2.2. Intracellular Distribution

Since compound **1** was the one presenting the best anti-cancer effect, it was selected for further studies regarding its possible mechanism of action.

In order to infer about the potential role of the sodium-dependent multivitamin transporters (SMVT) on the uptake of compound **1**, the intracellular distribution of this complex was determined in the presence or absence of free biotin. For that, MDA-MB-231 cells (that express SMVT [52]) were subjected to 1 h pre-incubation with free biotin before the addition of complex **1**. This assay was performed for a 3 h incubation period where cell death is negligible. We have simultaneously run the same experiment without the pre-incubation period with biotin, i.e., by adding complex **1** directly. Thus, to assess a possible competitive displacement for SMVT, cells were pre-treated with biotin competitor at a 1 mg/mL concentration (4.1 mM) in order to saturate the transporters and thus reduce their availability for complex **1**. Cytoskeletal, cytosol, membrane and nucleus fractions were extracted using a commercial kit as described in the Experimental Section.

**Figure 3:**
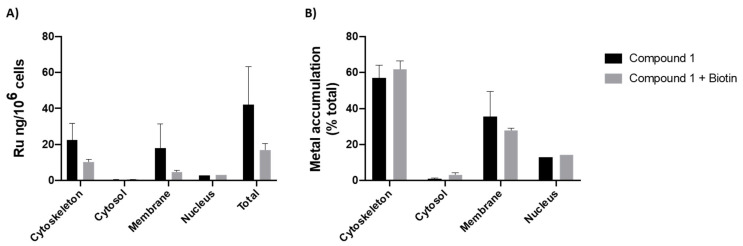
Cellular Ru distribution in the MDA-MB-231 cell line treated with compound **1** with or without a pre-incubation with biotin (1 mg/mL) at a concentration equivalent to the IC_50_ value found at 48 h challenge, 37 °C. Results are expressed in ng of Ru per million of cells (**A**) or in total percentage (**B**). Results are expressed as mean ± SD of two independent experiments.

As it can be observed in Figure 3, in the absence of biotin, complex **1** is mainly retained at the cytoskeleton (64%) and at the membrane (21%) of cancer cells, correlating well with that observed for the previously synthesized polymer–ruthenium conjugate, PMC78 [39]. As for the preliminary competitive study for SMVT, the Ru accumulation inside the cells after a 3 h challenge, is much lower after biotin pre-incubation (43.5 vs. 18.4 ng Ru/million cells), possibly indicating that the SMVT receptors are saturated with free biotin which decreases complex **1** uptake, thus suggesting that SMVT is the main transport route for this compound.

#### 3.2.3. Determination of the Type of Cell Death Induced by Compound **1**

**Figure 4:**
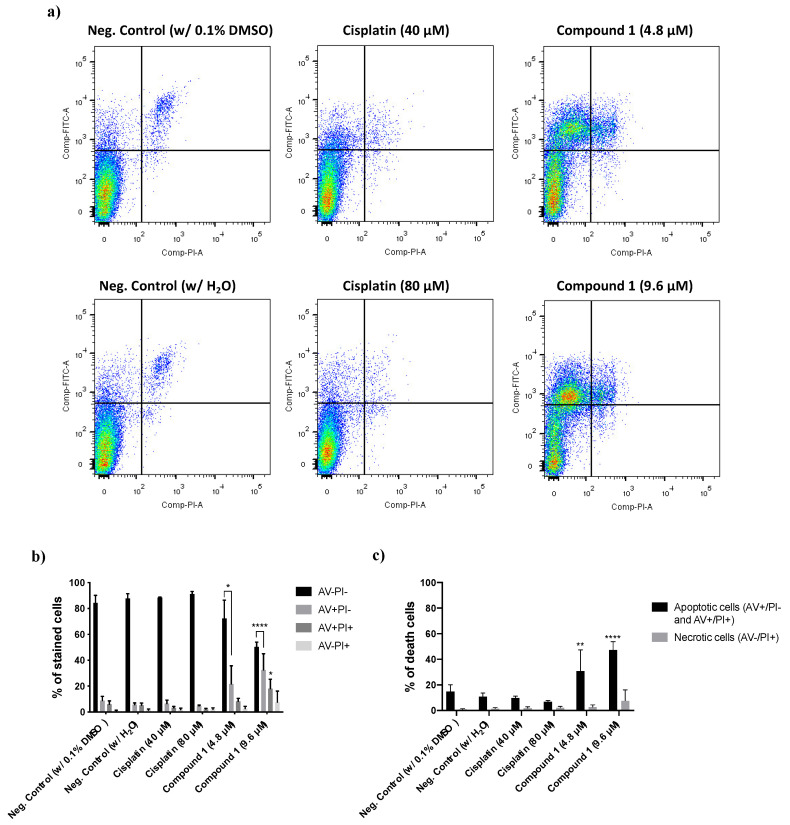
Apoptotic cell-death analysis by Annexin V-PI assay in MDA-MB-231 cells, using IC_50_ and 2 × IC_50_ concentrations for 48 h. (**a**) Representative histograms of MDA-MB-231 cell line double-stained with AV and PI. (**b**) Graphical representation of the Annexin V/PI dot plots. (**c**) Graphical representation of apoptotic (AV+/PI− and AV+/PI+) and necrotic (AV−/PI+) data analysis. * *p* ≤ 0.05; ** *p* ≤ 0.01; **** *p* ≤ 0.0001 compared with negative control (w/0.1% DMSO).

The cell death mechanism was assessed using Annexin V/PI assay by flow cytometry. MDA-MB-231 cells were incubated with compound **1**, for 48 h, at its IC_50_ and 2 × IC_50_ values, using cisplatin as a positive control. The results showed that the incubation with Ru compound **1** led to a significant increase in the percentage of Annexin V (AV+/PI- and AV+/PI+) stained cells in comparison to the negative control and cisplatin. These results indicate that the type of cell death induced by compound **1** is apoptosis instead of necrosis (Figure 4).

#### 3.2.4. Effect of Compound **1** in the Clonogenic Potential of MDA-MB-231 Breast Cancer Cells

**Figure 5:**
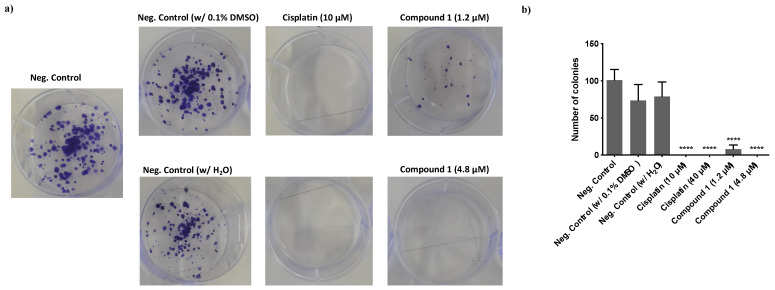
Colony formation capacity of MDA-MB-231 after 48 h of incubation with ¼ IC_50_ and IC_50_ of compound **1** in MDA-MB-231 cell line. (**a**) Representative images of colony formation assay in MDA-MB-231 cell line. (**b**) Graphical representation of clonogenic ability. **** *p* ≤ 0.0001 compared with negative control/w/0.1% DMSO).

The clonogenic ability of compound **1** was evaluated through colony formation assay. This technique allows for determining the cellular ability to survive to the exposure of an exogenous agent for a short period of time and produce colonies after that agent is removed. The colony formation assay gives us information about the true long-term effect that an agent has, even when it is not present, simulating in vitro what happens during cycles of chemotherapy. The MDA-MB-231 cell line was incubated with ¼ IC_50_ (1.2 μM) and IC_50_ (4.8 μM) values for 48 h, after which the medium was removed, and cells were maintained in culture for 8 days. The results showed that the Ru compound affected the ability to form colonies in a dose-dependent manner. The results were very similar to those of cisplatin in the condition with the IC_50._ In the case of ¼ IC_50_ of compound **1**, the formation of a small number of colonies was observed. Both concentrations of Ru compounds significantly prevented the formation of colonies (Figure 5).

#### 3.2.5. Evaluation of the Effect of Compound **1** on the Actin Cytoskeleton

**Figure 6:**
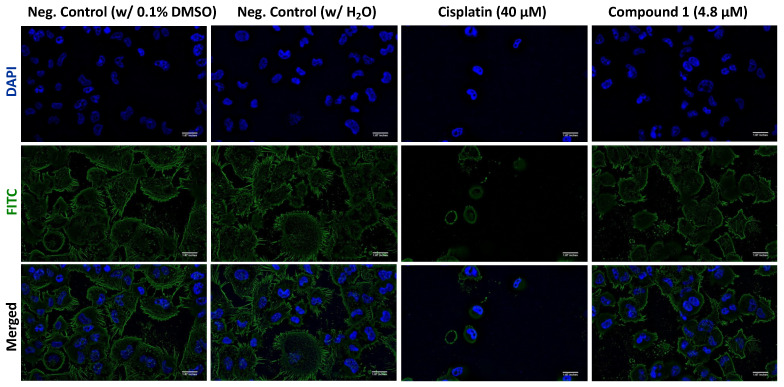
Compound **1** affects the cytoskeleton of MDA-MB-231 breast cancer cells. The analysis of F-actin staining was performed using the IC_50_ value of the compound, for 48 h. Representative images (×600) of DAPI (4′,6diamidino-2-phenylindole), Phalloidin-AlexaFluor^®^ 488 and merged were obtained by microscopy. The results were obtained from at least two independent experiments. Scale bar for all images is 1.67 inches.

The evaluation of the intracellular distribution of compound **1** in the MDA-MB-231 breast cancer-derived cell line demonstrated that this compound is mainly distributed in the cytoskeleton. This cellular fraction is essentially constituted by actin, and α- and β-tubulin proteins. Previous results from the group showed that Ru compounds induce changes in F-actin structure [28]. Taking this into consideration, we evaluated the effects of compound **1** in the actin cytoskeleton structure through observation of alterations in F-actin organization, using phalloidin. Cells were treated with IC_50_ concentrations of Ru compound, for 48 h, and then stained with Phalloidin-AlexaFluor^®^ 488 and DAPI (for staining the nucleic acids at the nucleus). The results showed that compound **1** affects the actin cytoskeleton, decreasing the size of the cells (Figure 6), although this effect is more visible with cisplatin. Besides that, cisplatin also led to a rounding of the cells and a decrease in the number of cells. In both conditions, we observed a decrease in the number of cell–cell adhesions, filipodia and lamellipodia like structures and intercellular contacts establishment. Compound **1** also led to the formation of evident actin belts at the cell periphery.

### 3.3. In Vivo Studies

As previously mentioned, compound **1** is the high-molecular weight version of **LCR134** (Figure 1C), designed having in view both active and passive targeting through the presence of biotin and PLA, respectively. Thus, we have performed preliminary testing of both compounds in vivo, aiming at clarifying whether the introduction of PLA is advantageous.

#### 3.3.1. Compounds’ Preparation

Since **LCR134** is not fairly soluble in DMSO and given the fact that the in vivo studies demand higher doses for the toxicity assessment, we used a modified patented cyclodextrin (Captisol^®^) to increase the administered dose. Therefore, a stock solution of 8 mg/mL of **LCR134** was prepared by first dissolving the complex in 30% DMSO and then slowly adding 20% of an aqueous solution of Captisol^®^. The final solution was placed in a mechanical shaker with continuous stirring overnight and was subsequently filtered with 20 μm filters and stored in aliquots at −80 °C.

As for the polymeric complex **1**, a stock solution of 8 mg/mL in 30% DMSO/70% MQ water was prepared by first dissolving the compound in DMSO and then slowly adding MQ water. The solution was filtered (40 μm filters) and diluted to the corresponding tested concentrations. The instantaneous diffusion of the organic solvent into the aqueous solution resulted in the formation of polymeric NPs as characterized by their hydrodynamic diameter of ca. 77 ± 10 nm with a polydispersity index (PdI) of 0.26 (Figure S27). However, the formation of aggregates with time was also observed. Yet, since the purpose of the in vivo studies was just a preliminary assessment on the compound’s potential, no further optimizations were performed.

#### 3.3.2. Toxicology Assay

The main goal of this initial toxicity assay was to evaluate the toxic impact of the drug and to find the maximum tolerated dose in mice. For that, we treated the mice (3–4 per group), twice a week, for 15 days, by IP injection with four different drug dosages (0.5 mg/kg, 1.5 mg/kg, 3 mg/kg and 4 mg/kg) and followed the design scheme represented on Figure S28.

**Figure 7:**
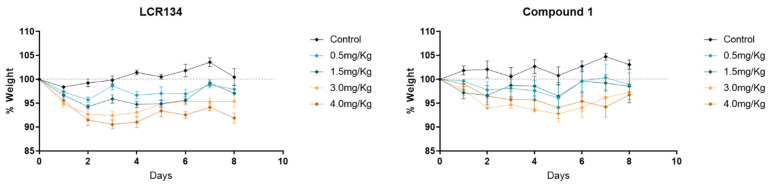
Effect of **LCR134** and compound **1** in animal health, measured in terms of body weight. Body weight was measured once a day during toxicology assay.

**Figure 8:**
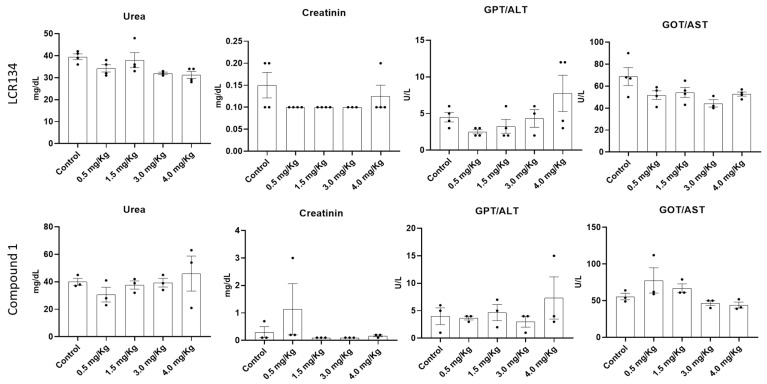
Quantification of kidney and liver function. Urea, creatinine, AST and ALT enzymes concentrations detected in the plasma of the **LCR134** and compound **1** treated mice. Each dot represents one tested blood sample per animal.

As observed in Figure 7, **LCR134** and compound **1** showed a tendency towards a decrease in body weight. Although not significant, it was interesting to observe that **LCR134** had a more pronounced effect on mice weight when compared with compound **1**. These results were further confirmed by the quantification of liver and kidney functions, through analysis of AST and ALT enzymes and urea and creatinine waste products, respectively (Figure 8). Although not significant, both **LCR134** and compound **1** showed lower creatinine levels for the three higher concentrations and concomitantly, the highest 4 mg/kg concentration also showed a tendency for an increase in ALT function, usually associated with liver toxicity.

These results show that the mice were tolerant to the maximum tested dose for the ruthenium-based compounds **LCR134** and **1** (4 mg/kg), although it already shows some liver toxicity. Considering this data, further experiments will be useful to determine the maximum tolerated dose, as well as to better define the impact of ruthenium-based compounds in hepatic function.

#### 3.3.3. Tumour Growth

The next step was to evaluate the potential therapeutic effect of **LCR134** and compound **1** in tumorigenesis, using the maximum tolerated dose of 4 mg/kg, in a breast cancer orthotopic model, and following the experimental design depicted in Figure S29, detailed in the experimental section.

**Figure 9:**
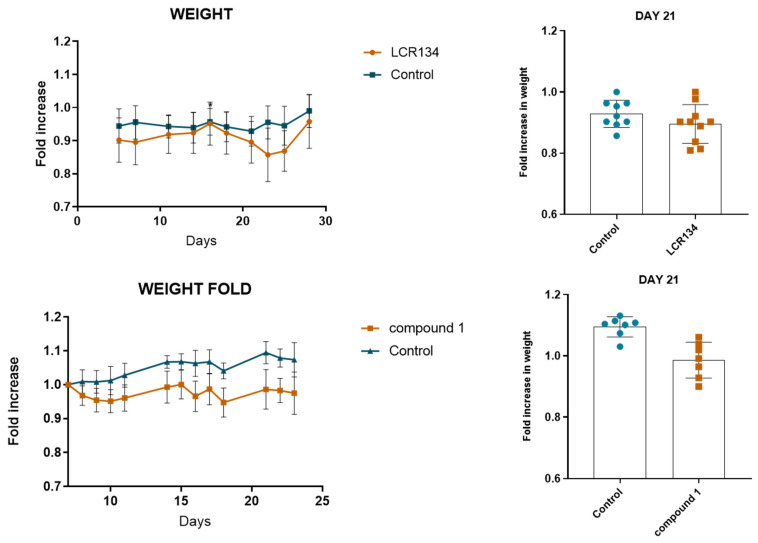
Effect of compounds in animal health, measured in terms of body weight. Body weight was measured twice a week. Each dot and square represent one animal.

**Figure 10:**
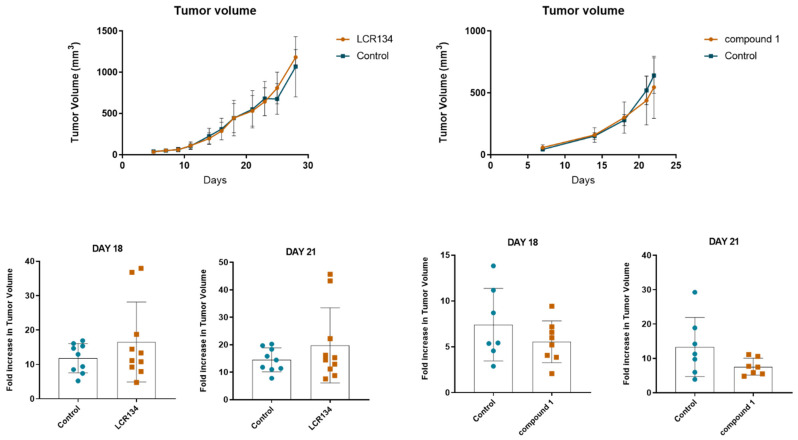
Effect of ruthenium-based compounds **LCR134** and **1** in tumor volume evolution of MDA-MB-231 orthotopically inoculated in the mammary fat pad of N:NIH(s)II-nu/nu nude female mice from day 1 to day 23. Each dot and square represent one animal.

Our results show that there are no significant differences in the mice weight (Figure 9) and in tumor volume (Figure 10) between the control and the treated groups (**LCR134** and compound **1**). Nevertheless, for compound **1** a tendency towards a decrease in tumor growth was clearly seen. These data demonstrate that, under these experimental conditions, the **LCR134** and compound **1** did not promote a significant toxicity to the mice, nor significantly affected the primary tumor growth in mice. Still, a more pronounced effect on tumor growth was achieved with compound **1**, suggesting that it has improved in vivo functions when compared with **LCR134**. Further studies such as the impact on metastasis prevention and/or treatment and the optimization of compound’s formulation to allow higher treatment doses would be useful to really determine its effect in vivo.

## 4. Conclusions

A family of four polymer-ruthenium conjugates was newly synthesized and completely characterized by spectroscopic and analytical techniques. Among them, compound **1**, [Ru(η^5^-Cp)(P(C_6_H_5_)_3_)(bipy-PLA-biotin)]^+^, presented the best cytotoxicity against the triple-negative breast cancer cells MDA-MB-231 and was selected for further studies. We showed that internalization via SMVT receptors was probably the major transport route for cells uptake of compound **1,** indicating that biotin is playing its role for the active targeting. Regarding its mechanism of action, compound **1** causes apoptosis, affects proliferation by decreasing the formation of cancer cell colonies and interferes with cells cytoskeleton. Ruthenium cellular distribution indicates that compound **1** targets are probably located at cell cytoskeleton and/or cell membrane.

For the preliminary in vivo assays in N: NIH(S)II-nu/nu mice, besides testing compound **1**, we included **LCR134**, aimed only at active targeting, to check the influence of the PLA chains on the activity. Even though some formulation optimization will be needed for the future, compound **1** shows a tendency to control tumor growth, contrarily to **LCR134**. We could establish a pre-clinical proof of concept for compound **1** in the treatment of triple-negative breast cancer.

To sum up, compound **1** shows promising anti-cancer effects that should be further explored in the frame of triple-negative breast cancer therapy.

## Figures and Tables

**Table 1:** Experimental data for the bipy-PLA-OH synthesized.

	Yield (%) ^a^	*DP* ^b^	*M*_n,NMR_^c^ (g/mol)
Bipy-PLA-OH (**PLA1**)	95	58	4392.2
Bipy-PLA-OH (**PLA2**)	88	66	4968.2
Bipy-PLA-OH (**PLA3**)	86	62	4680.2

^a^ After purification; ^b^ Degree of polymerization (DP) after purification of the polymer was calculated by ^1^H-NMR dividing the relative proton peak intensity of the bipyridine moiety (H_1_ proton) to that of the repeating polymer chain unit of interest (H_A_ proton) according to [48]. ^c^ Number-average molecular weight Mn determined by ^1^H-NMR.

**Table 2:** Degree of polymerization (DP) for the 2,2′-Bipyridine-4,4′-PLA-Biotin (L1) synthesized.

	DP ^a^
**L1_PLA1**	68
**L1_PLA2**	60
**L1_PLA3**	58

^a^ Degree of polymerization (DP) after purification of the polymer was calculated by ^1^H-NMR dividing the relative proton peak intensity of the bipyridine moiety (H_1_ proton) to that of the repeating polymer chain unit of interest (H_A_ proton) according to [48].

**Table 3:** Experimental data regarding the degree of polymerization (DP) and molecular weight of complexes **1**–**4**.

	DP ^a^	Mn (^1^H-NMR) ^b^
Complex **1**	70	6233.9 g/mol
Complex **2**	64	5941.6 g/mol
Complex **3**	42	4319.4 g/mol
Complex **4**	56	5296.0 g/mol

^a^ Degree of polymerization (DP) after purification of the polymer was calculated by ^1^H-NMR dividing the relative proton peak intensity of the bipyridine moiety (H_1_ proton) to that of the repeating polymer chain unit of interest (H_A_ proton) according to [48]. ^b^ Number-average molecular weight *M*_n_ determined by ^1^H-NMR.

**Table 4:** IC_50_ Values (μM) for Complexes **1**−**4**, **LCR134, LCR205, LCR234, CDDP**, and bipy-PLA-biotin based ligand (**L1**) at 48 h incubation, in MDA-MB-231 breast cancer cell line. Values are mean ± SD of three independent experiments.

Compound	MDA-MB-231 (μM)
[Ru(η^5^-Cp)(P(C_6_H_5_)_3_)(bipy-biotin)]^+^ (**LCR134**)	3.2 ± 1.1 [23]
[Ru(η^5^-Cp)(P(C_6_H_5_)_3_)(bipy-PLA-biotin)]^+^ (**1**)	2.3 ± 0.1
[Ru(η^5^-Cp)(P(C_6_H_4_F)_3_)(bipy-biotin)]^+^ (**LCR205**)	7.1 ± 0.6 [23]
[Ru(η^5^-Cp)(P(C_6_H_4_F)_3_)(bipy-PLA-biotin)]^+^ (**2**)	14.6 ± 0.4
[Ru(η^5^-Cp)(P(C_6_H_4_OCH_3_)_3_)(bipy-biotin)]^+^ (**LCR234**)	4.0 ± 0.2 [23]
[Ru(η^5^-Cp)(P(C_6_H_4_OCH_3_)_3_)(bipy-PLA-biotin)]^+^ (**3**)	6.5 ± 0.3
[Ru(η^5^-MeCp)(P(C_6_H_5_)_3_)(bipy-PLA-biotin)]^+^ (**4**)	3.4 ± 0.1
CDDP	40 ± 3.7
bipy-PLA-biotin (**L1**)	>100

## Supplementary Materials

The following supporting information can be downloaded at: https://www.mdpi.com/article/10.3390/pharmaceutics14071388/s1, Figures S1–S5: FT-IR spectra; Figures S6–S17: NMR spectra; Figures S18–S21: MALDI-TOF MS spectra; Figures S22–S25: Partial HR-MS spectra; Figure S26: Dose-response curves; Figure S27: Hydrodynamic diameter distribution (obtained by DLS) of complex **1**; Figure S28: Experimental design of toxicology assay performed; Figure S29: Experimental design of tumor growth assay.
