# Supplementary material for: Biotinylated Polymer-Ruthenium Conjugates: In Vitro and In Vivo Studies in a Triple-Negative Breast Cancer Model
Source: Pharmaceutics. 2022 Jun 30;14(7):1388. doi: 10.3390/pharmaceutics14071388 (PMC9315599; doi:10.3390/pharmaceutics14071388)
Supplement: Supplementary file 1 [file pharmaceutics-14-01388-s001.zip › pharmaceutics-1714009-supplementary.pdf]

Supporting Information

Biotinylated Polymer-Ruthenium Conjugates: In Vitro and In Vivo Studies in a Triple-Negative Breast Cancer Model

Leonor Côrte-Real [1], Ana Rita Brás [1,2,3], Adhan Pilon [1], Nuno Mendes [4], Ana Sofia Ribeiro [4], Tiago D. Martins [5], José Paulo S. Farinha [5], M. Conceição Oliveira [5], Fátima Gärtner [4], M. Helena Garcia [1], Ana Preto [2,3] and Andreia Valente [1,*]

[1] Centro de Química Estrutural, Institute of Molecular Sciences and Departamento de Química e Bioquímica, Faculdade de Ciências, Universidade de Lisboa, Campo Grande, 1749-016 Lisboa, Portugal; ldcortereal@fc.ul.pt (L.C.-R.); pg31015@alunos.uminho.pt (A.R.B.); adpilon@fc.ul.pt (A.P.); mhgarcia@fc.ul.pt (M.H.G.)

[2] Centre of Molecular and Environmental Biology, Department of Biology, University of Minho, Campus de Gualtar, 4710-057 Braga, Portugal; apreto@bio.uminho.pt

[3] Institute of Science and Innovation for Bio-Sustainability, University of Minho, Campus de Gualtar, Edifício 18, 4710-057 Braga, Portugal

[4] i3S—Instituto de Investigação e Inovação em Saúde, Universidade do Porto, 4200-135 Porto, Portugal; nmendes@ipatimup.pt (N.M.); aribeiro@ipatimup.pt (A.S.R.); fgartner@ipatimup.pt (F.G.)

[5] Centro de Química Estrutural, Institute of Molecular Sciences and Department of Chemical Engineering, Instituto Superior Técnico, Universidade de Lisboa, Av. Rovisco Pais, 1049-001 Lisboa, Portugal; tiagodmartins@tecnico.ulisboa.pt (T.D.M.); farinha@tecnico.ulisboa.pt (J.P.S.F.); conceicao.oliveira@tecnico.ulisboa.pt (M.C.O.)

* Correspondence: amvalente@fc.ul.pt

FT-IR spectra

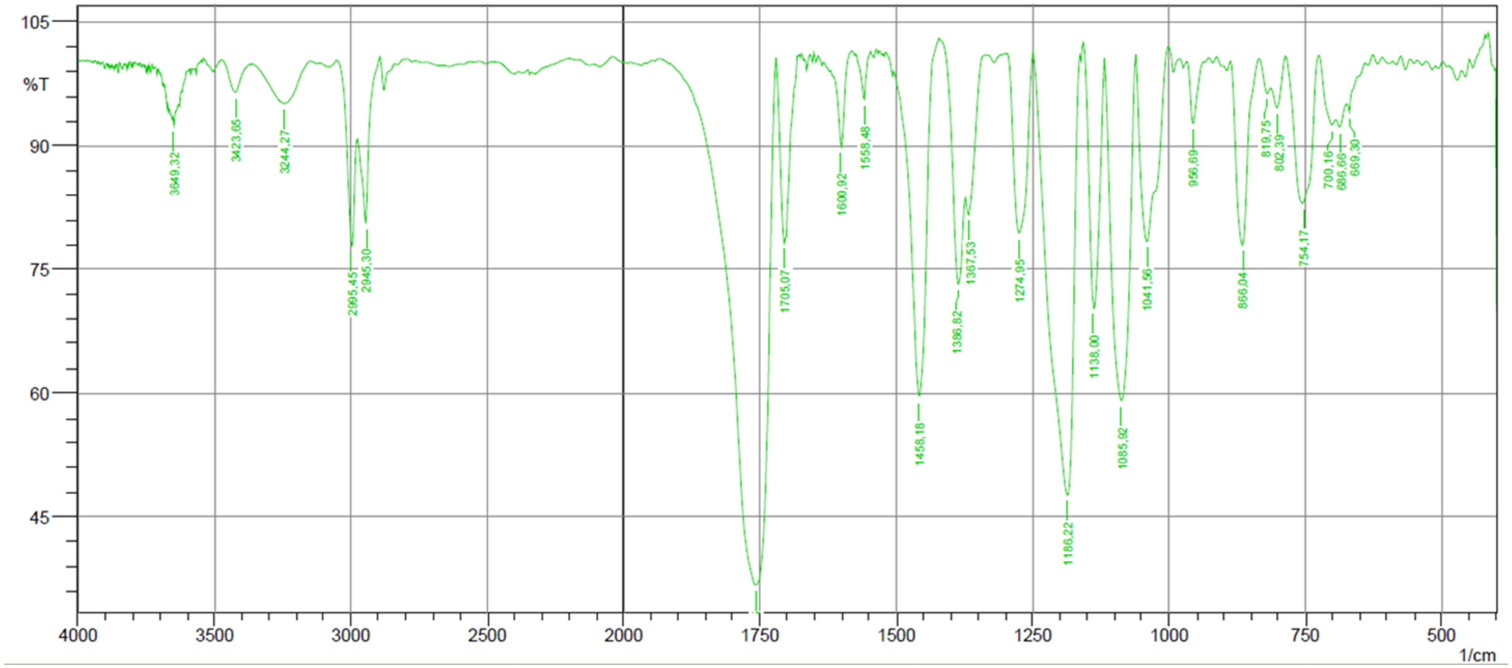

Figure S1. FT-IR spectrum of Ligand L1.

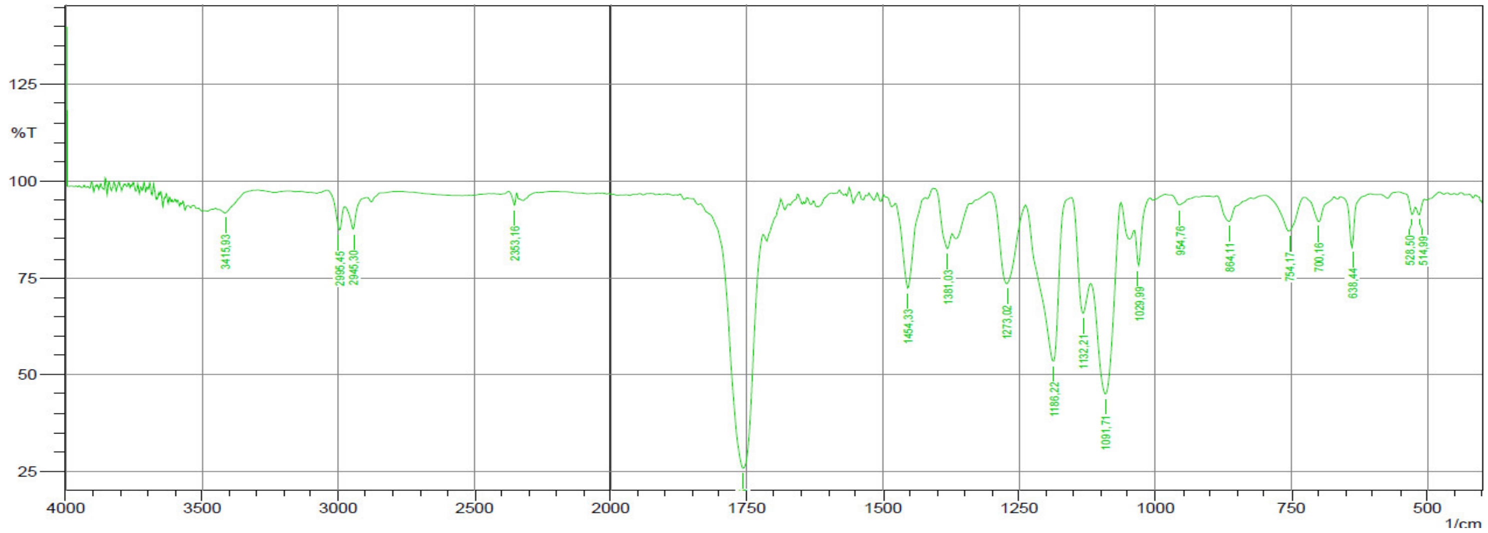

Figure S2. FT-IR spectrum of Complex **1**.

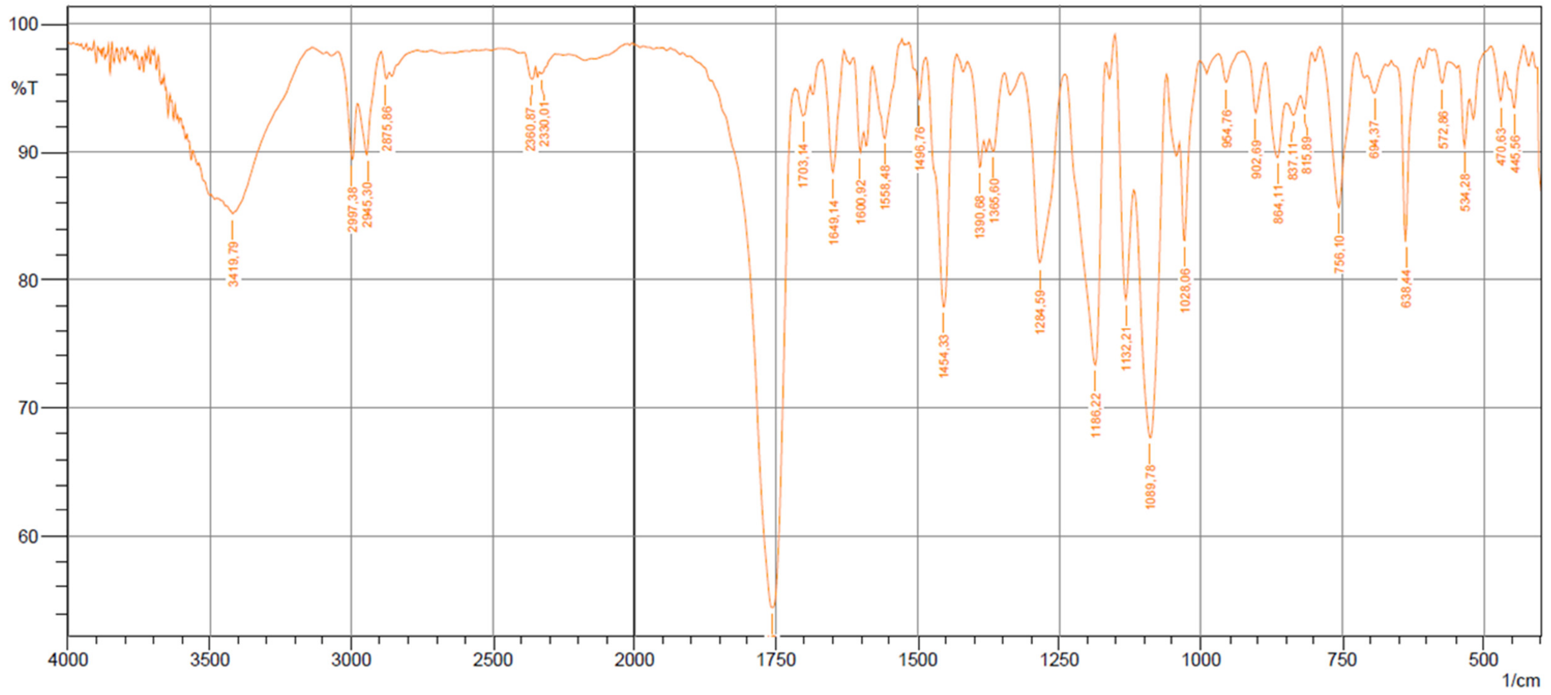

Figure S3. FT-IR spectrum of complex **2**.

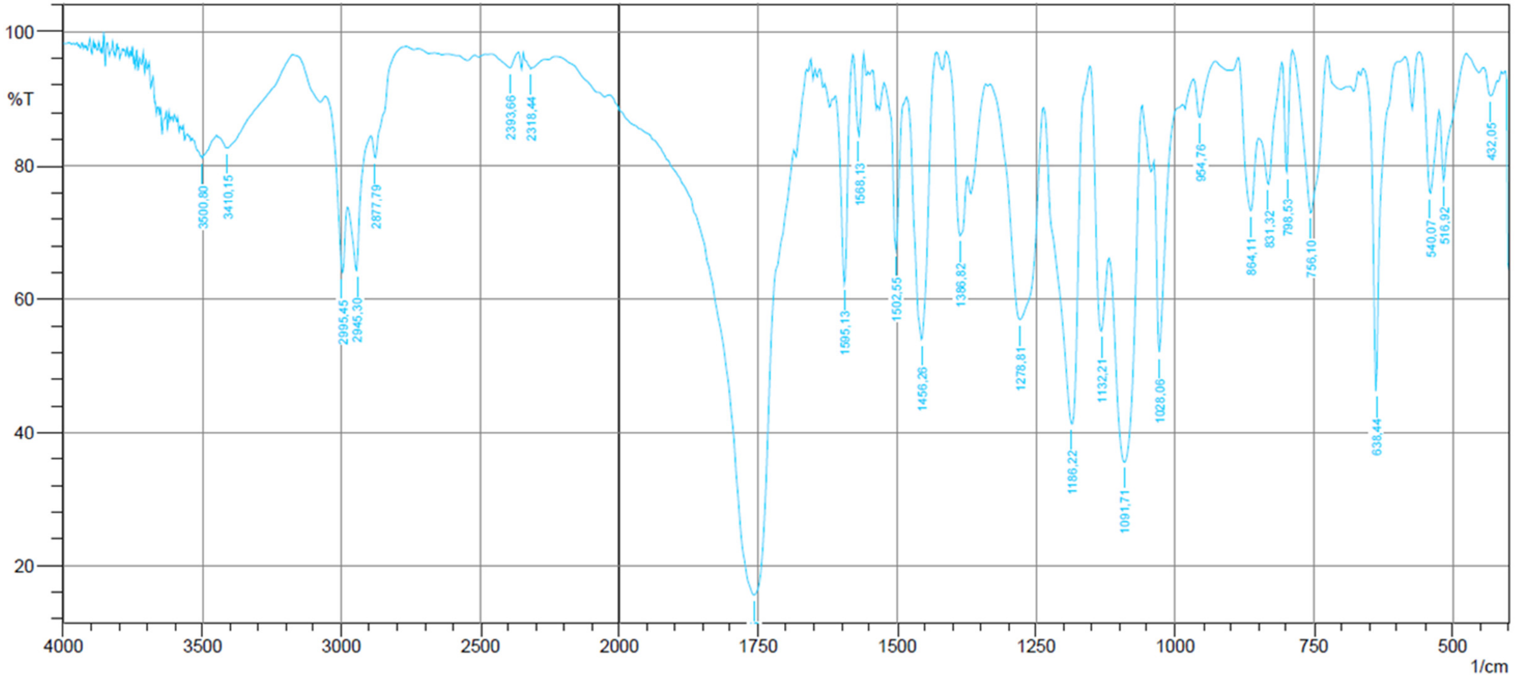

Figure S4. FT-IR spectrum of complex **3**.

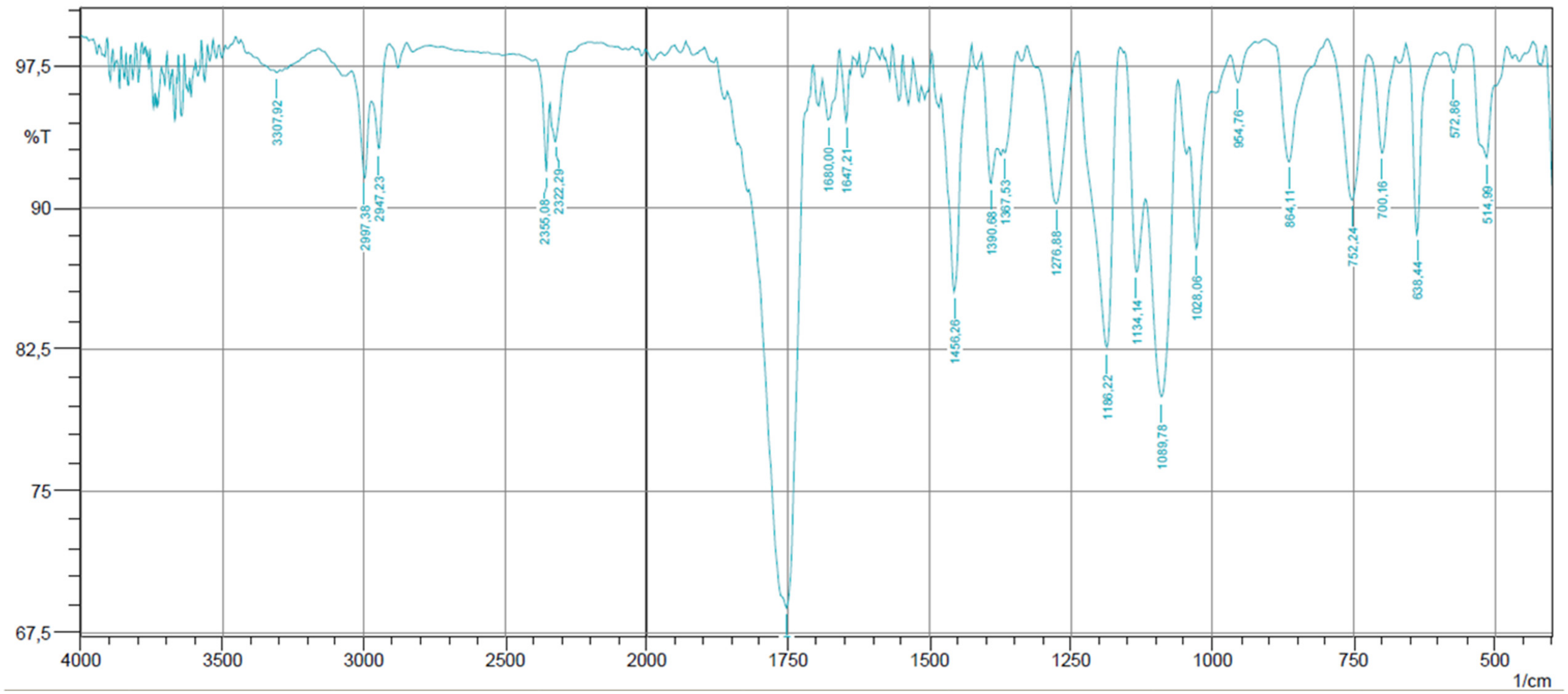

Figure S5. FT-IR spectrum of complex **4**.

NMR spectra

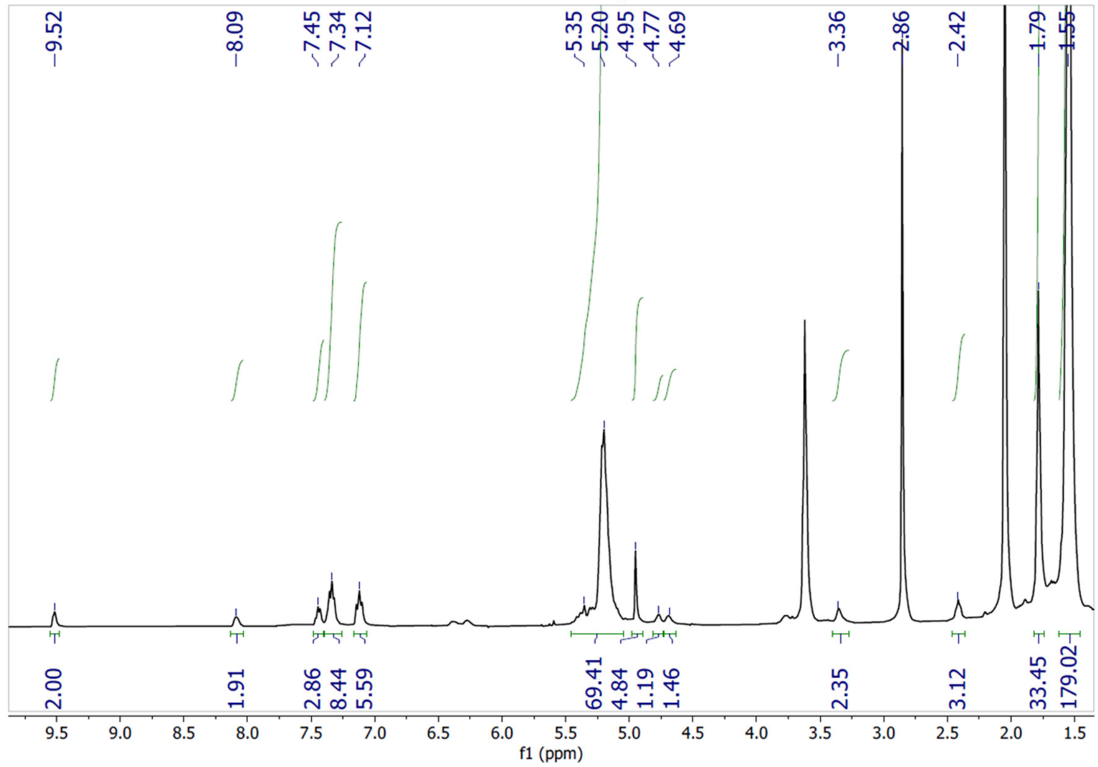

Figure S6. ^{1}H-NMR spectrum of complex **1** in acetone-d_6 at 298 K.

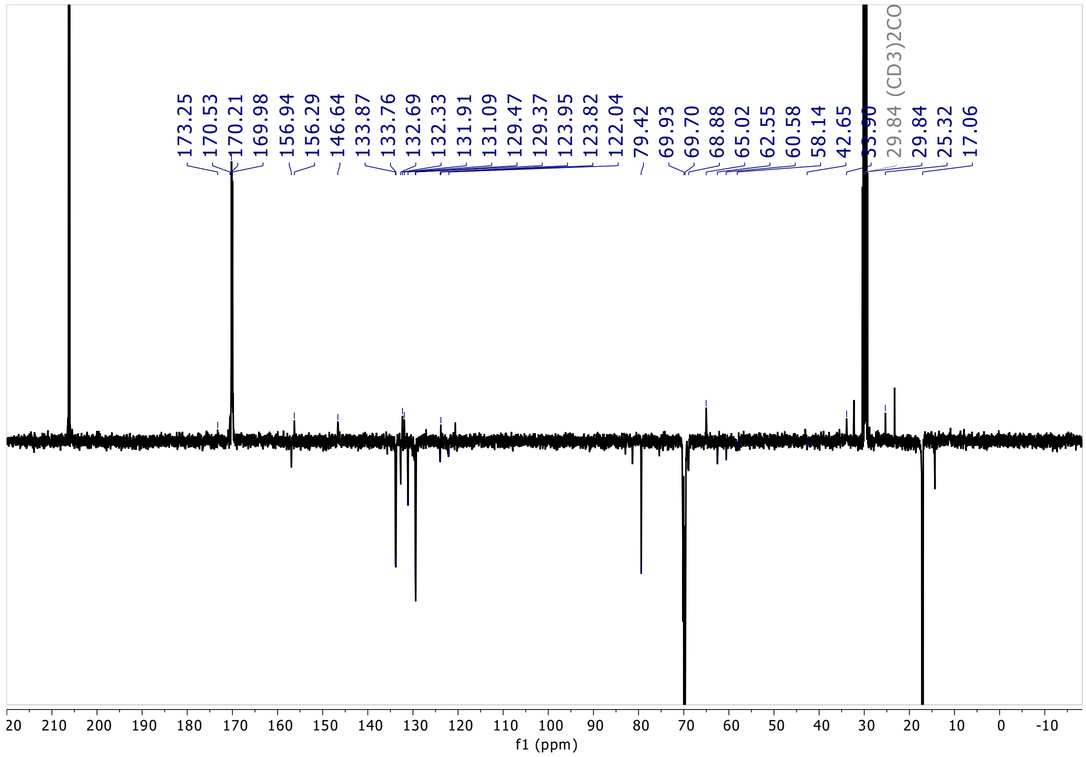

Figure S7. APT $^{13}C\{^{1}H\}$-NMR spectrum of complex **1** in acetone-d_6 at 298 K.

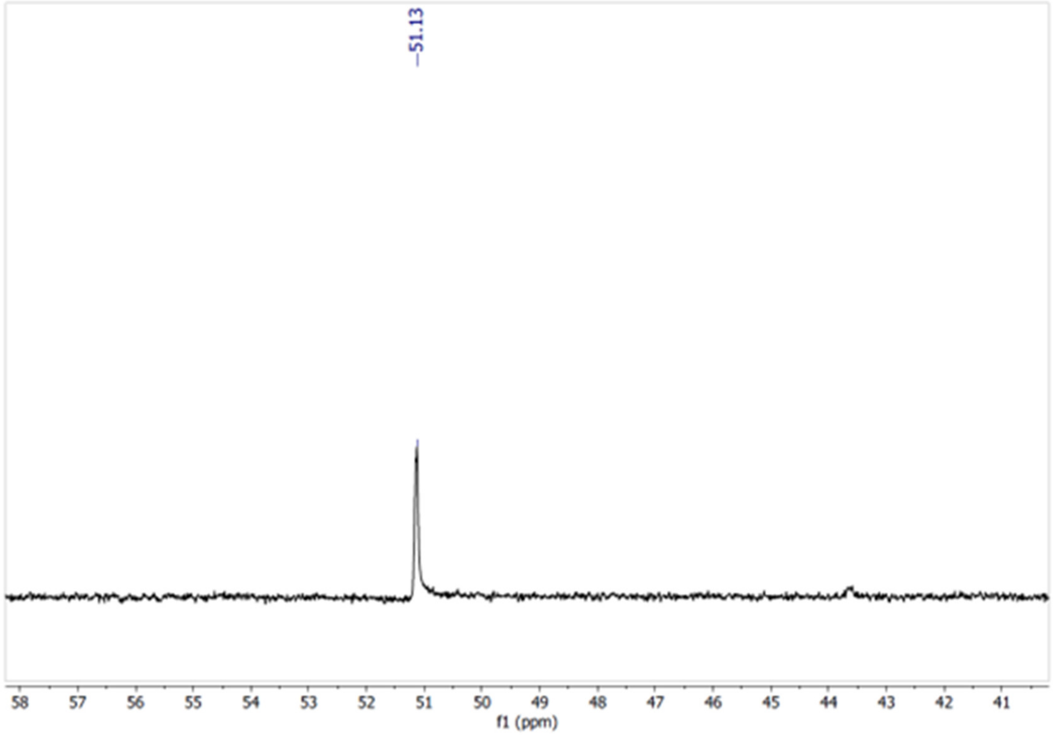

Figure S8. $^{31}P\{^{1}H\}$-NMR spectrum of complex **1** in acetone-d_6 at 298 K.

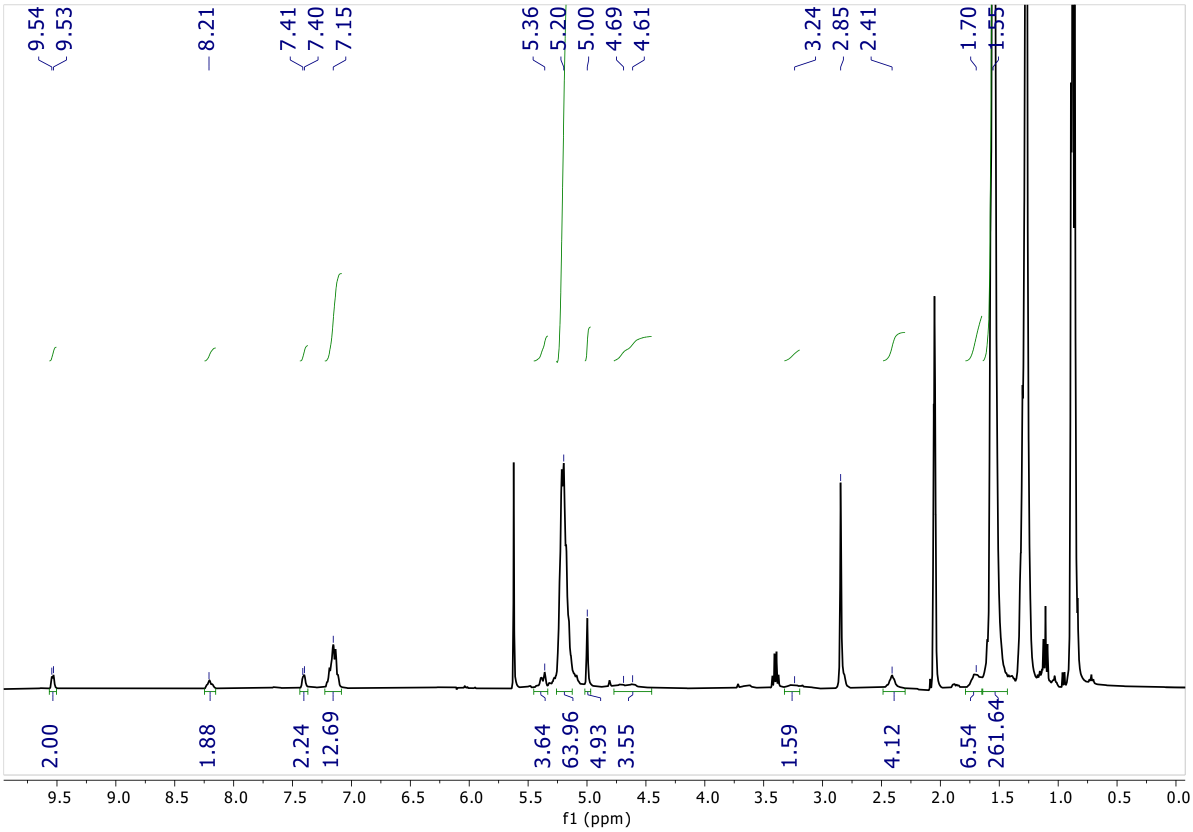

Figure S9. ^{1}H-NMR spectrum of complex **2** in acetone-d_6 at 298 K.

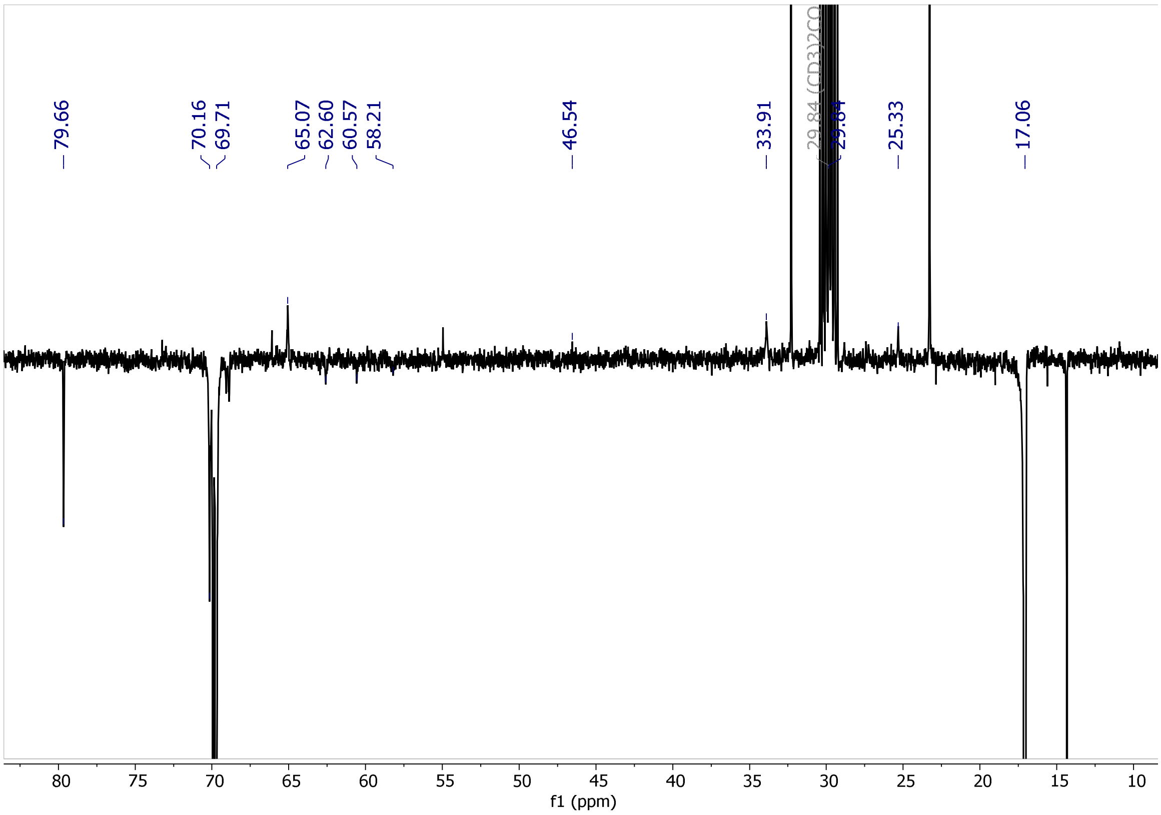

Figure S10. APT $^{13}C\{^{1}H\}$-NMR spectrum of complex **2** in acetone-d_6 at 298 K.

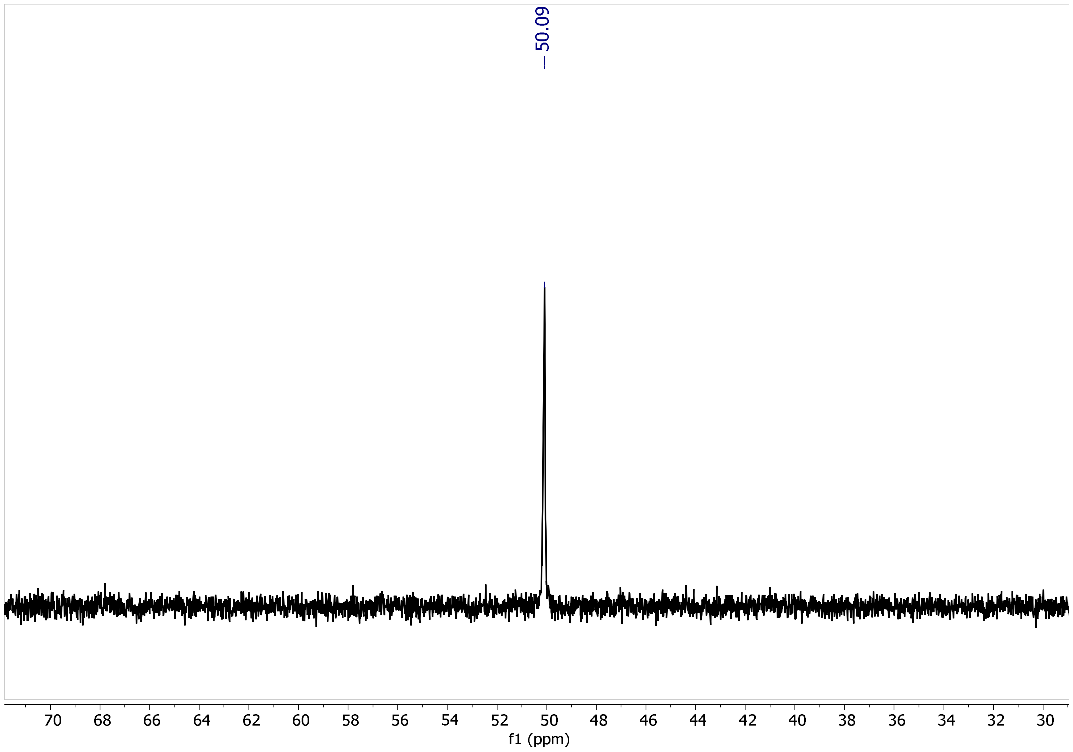

Figure S11. $^{31}P\{^{1}H\}$-NMR spectrum of complex **2** in acetone-d_6 at 298 K.

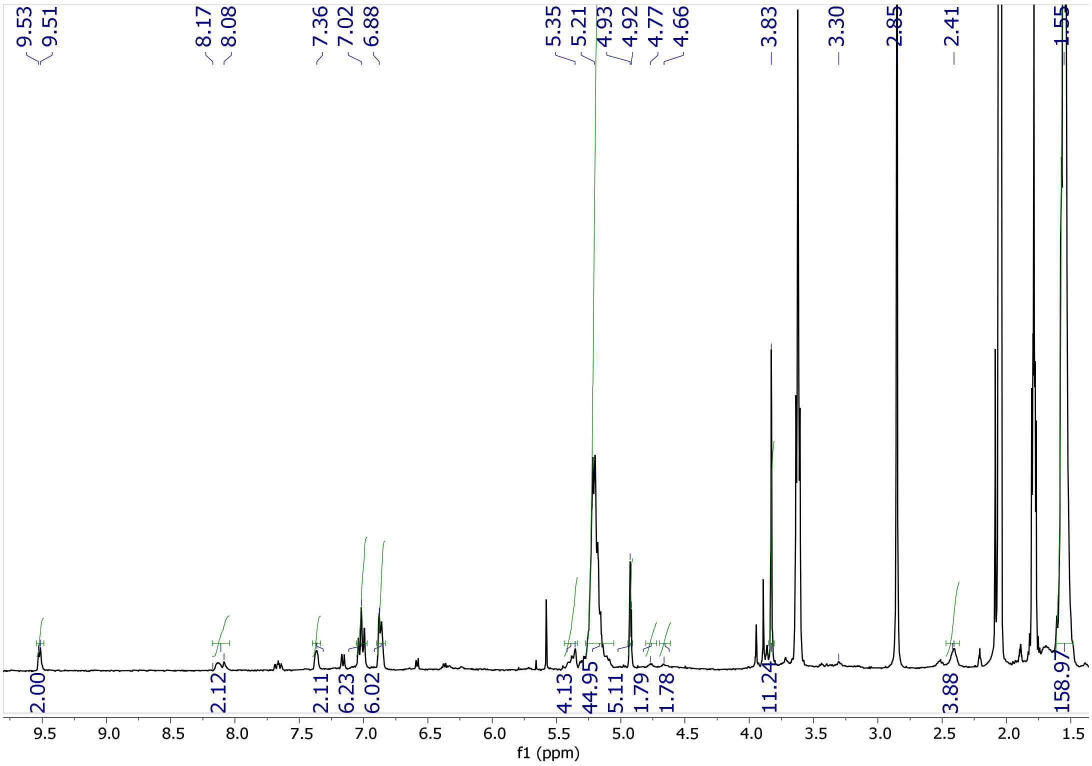

Figure S12. ^{1}H-NMR spectrum of complex **3** in acetone-d_6 at 298 K.

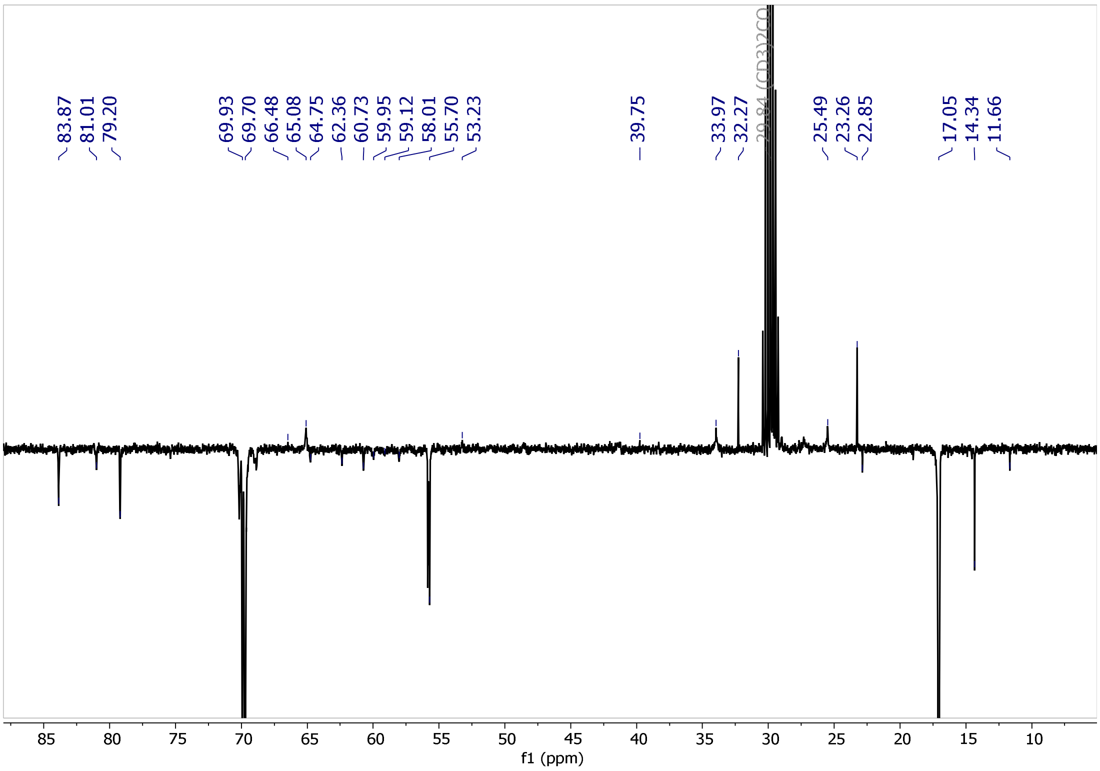

Figure S13. APT $^{13}C\{^{1}H\}$-NMR spectrum of complex **3** in acetone-d_6 at 298 K.

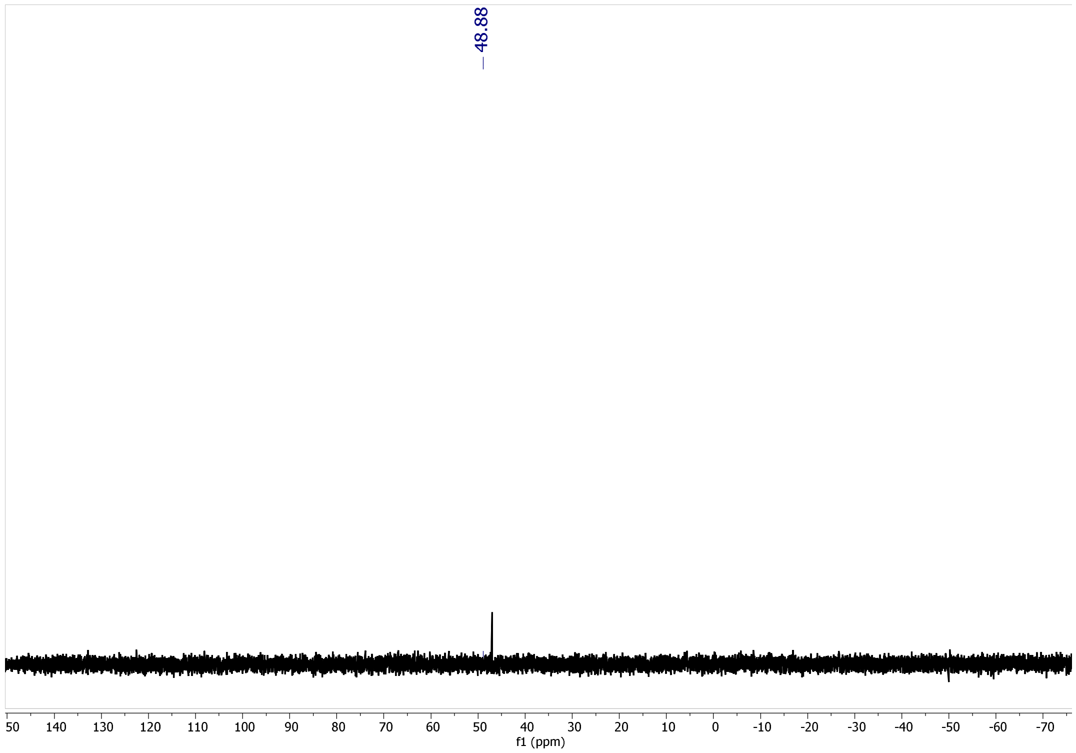

Figure S14. $^{31}P\{^{1}H\}$-NMR spectrum of complex **3** in acetone-d_6 at 298 K.

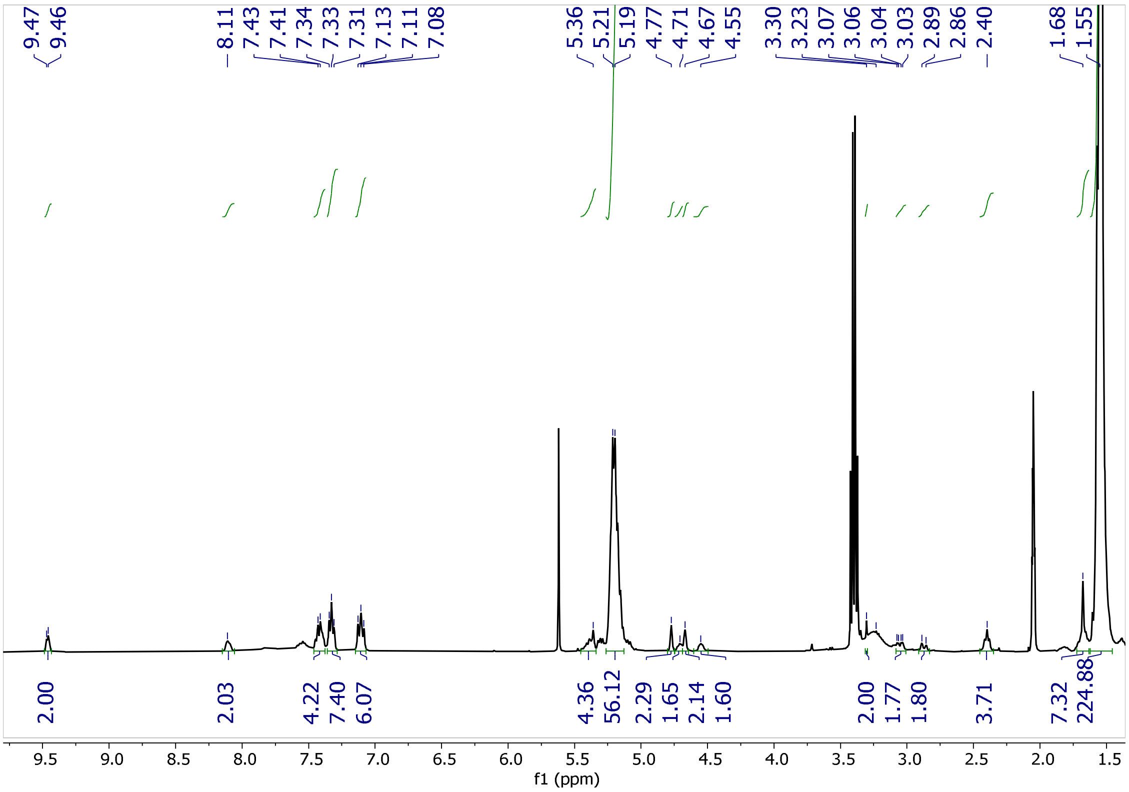

Figure S15. ^{1}H-NMR spectrum of complex **4** in acetone-d_6 at 298 K.

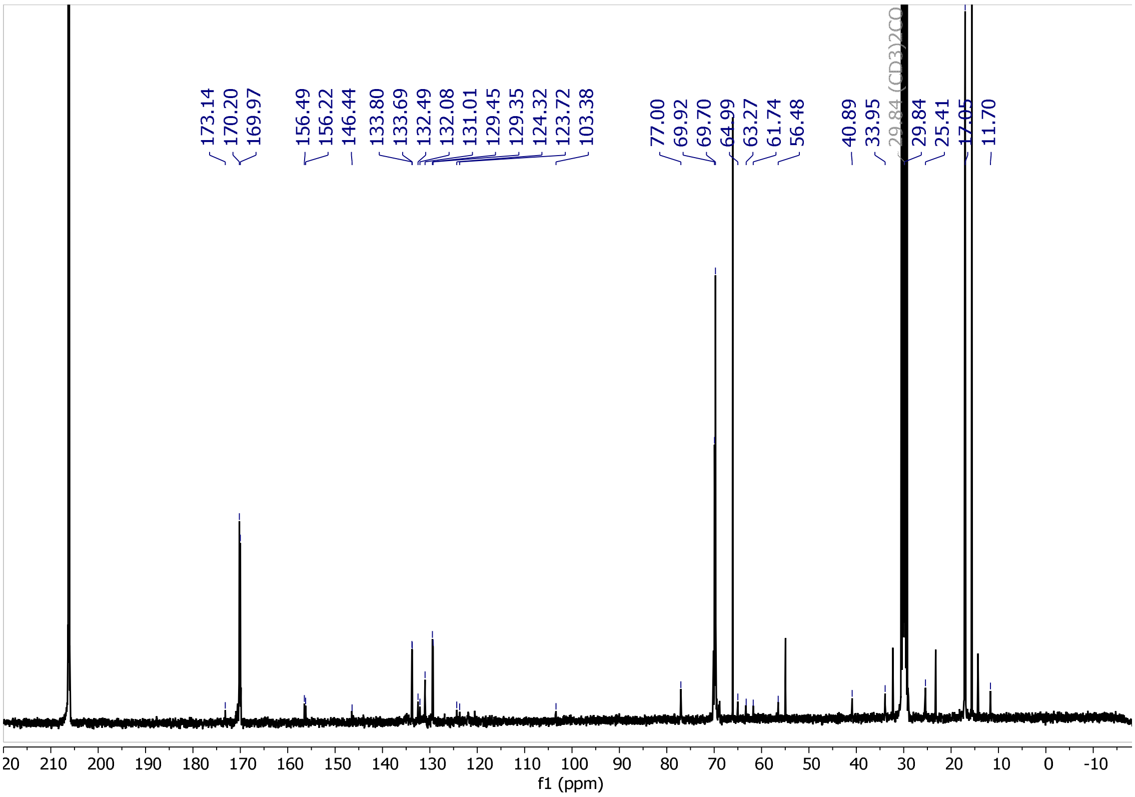

Figure S16. $^{13}C\{^{1}H\}$-NMR spectrum of complex **4** in acetone-d_6 at 298 K.

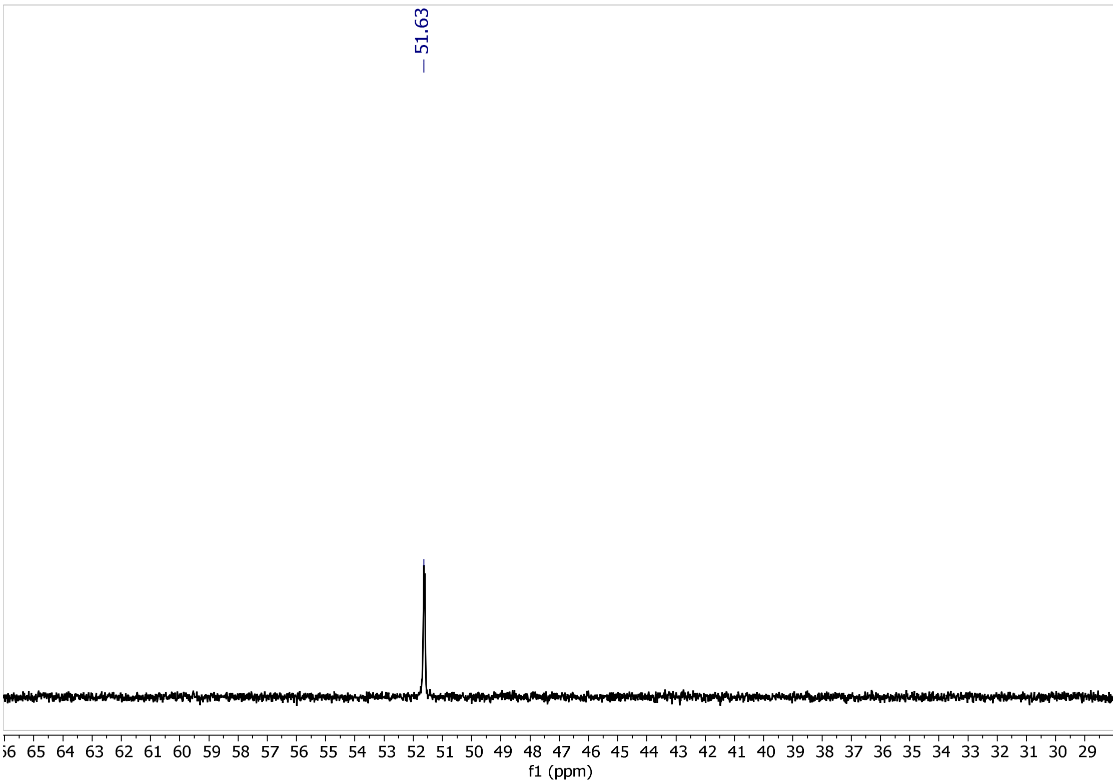

Figure S17. $^{31}P\{^{1}H\}$-NMR spectrum of complex **4** in acetone-d_6 at 298 K.

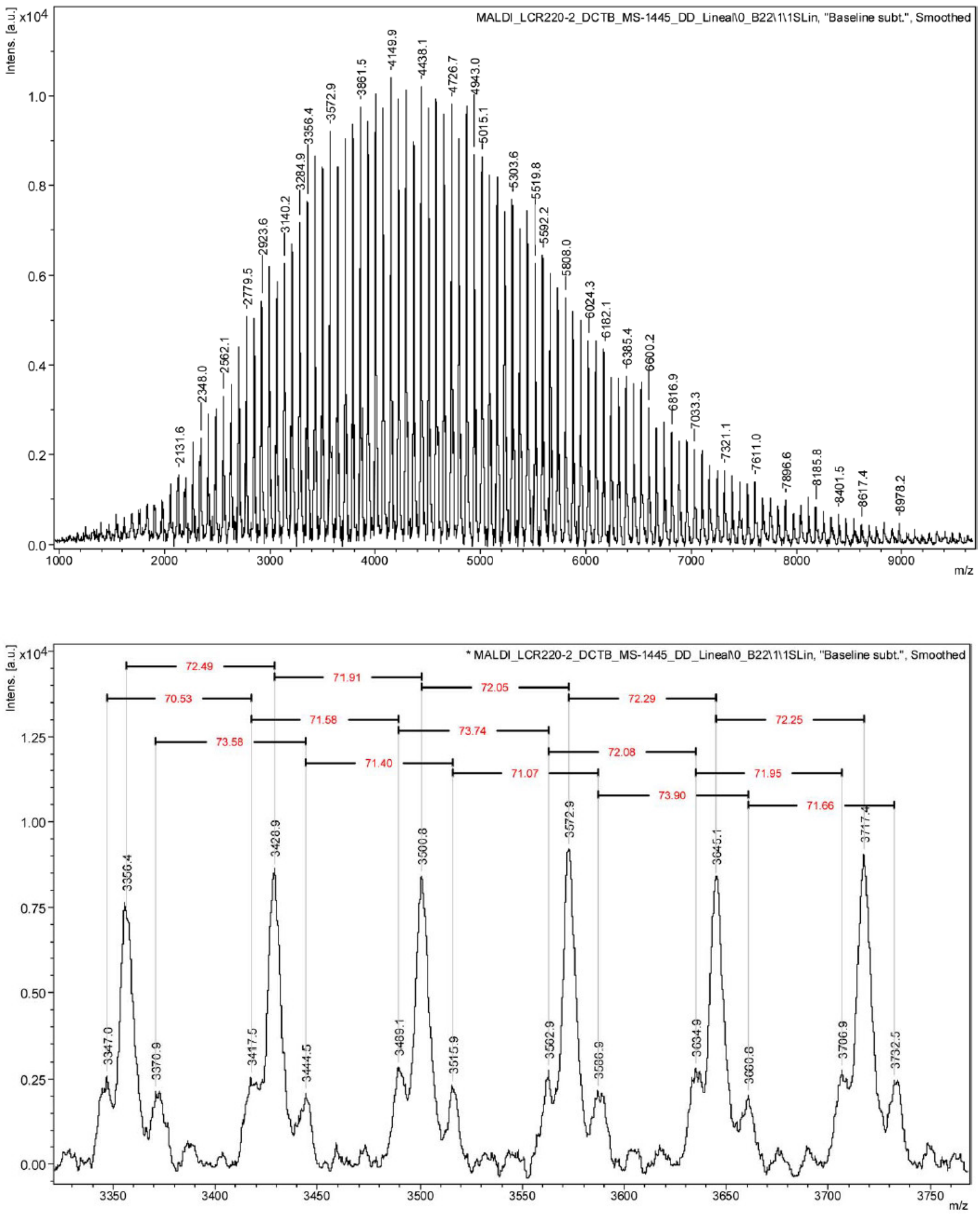

Figure S18. MALDI-TOF MS analysis of Compound **1.**

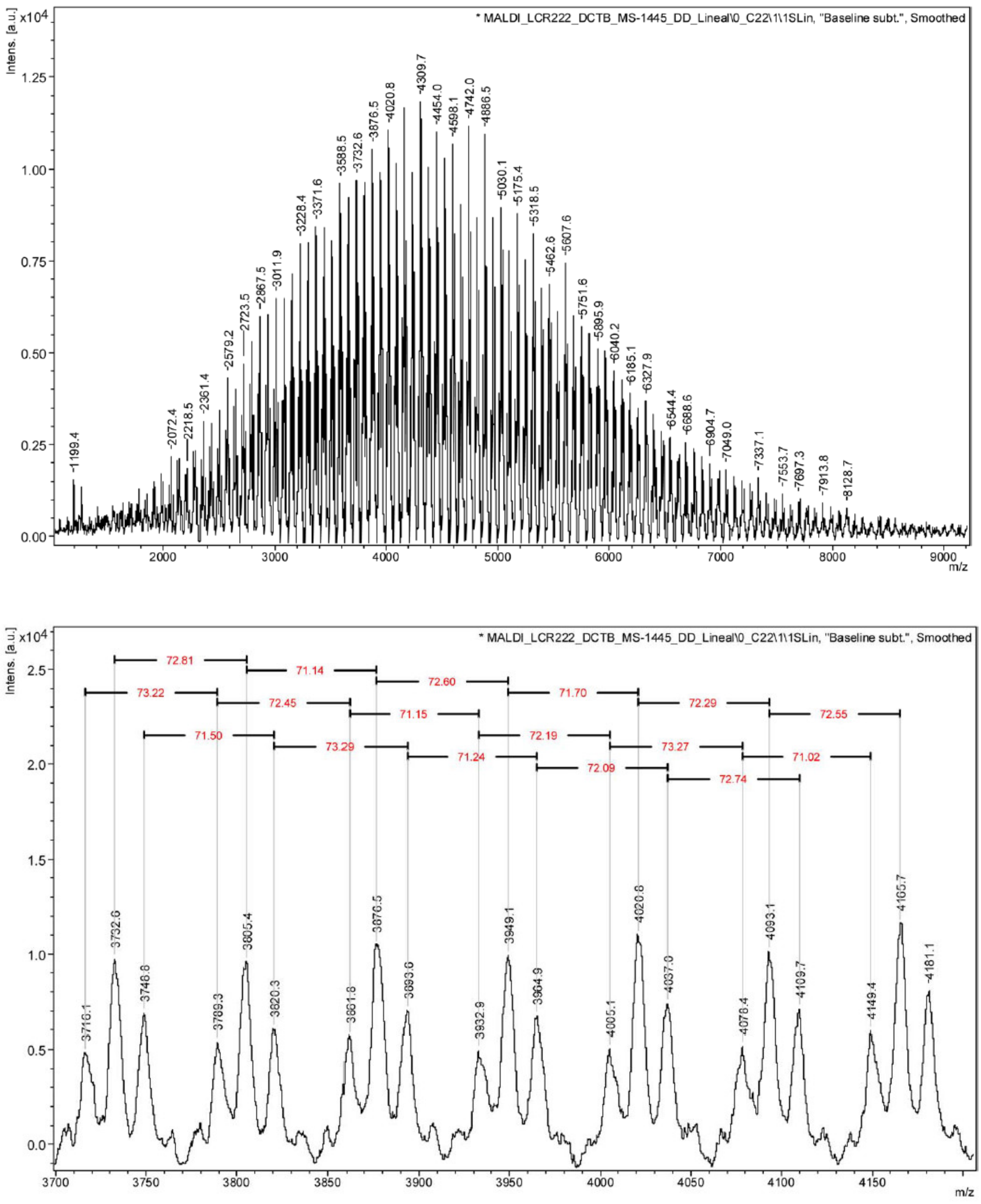

Figure S19. MALDI-TOF MS analysis of Compound **2.**

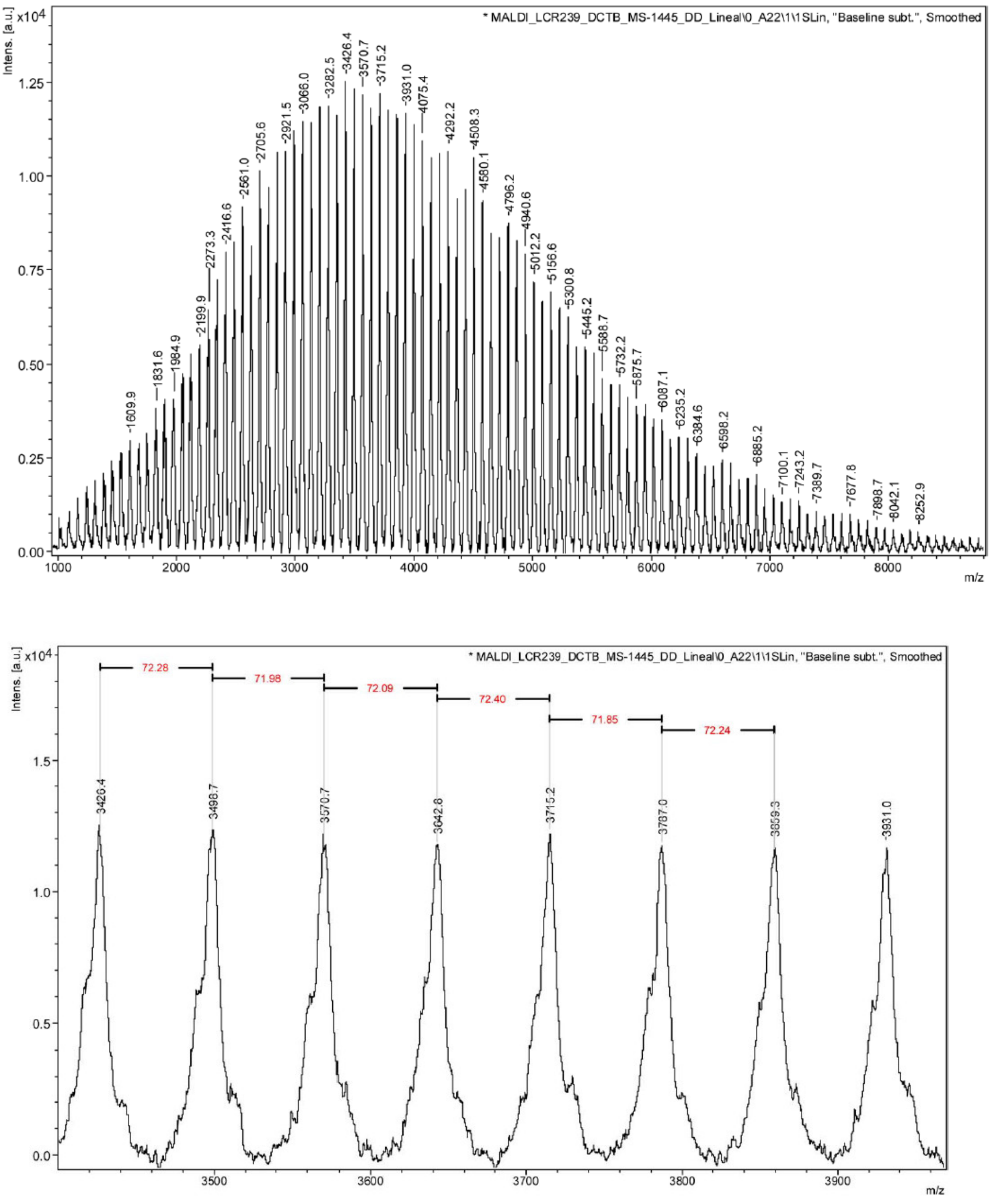

Figure S20. MALDI-TOF MS analysis of Compound **3.**

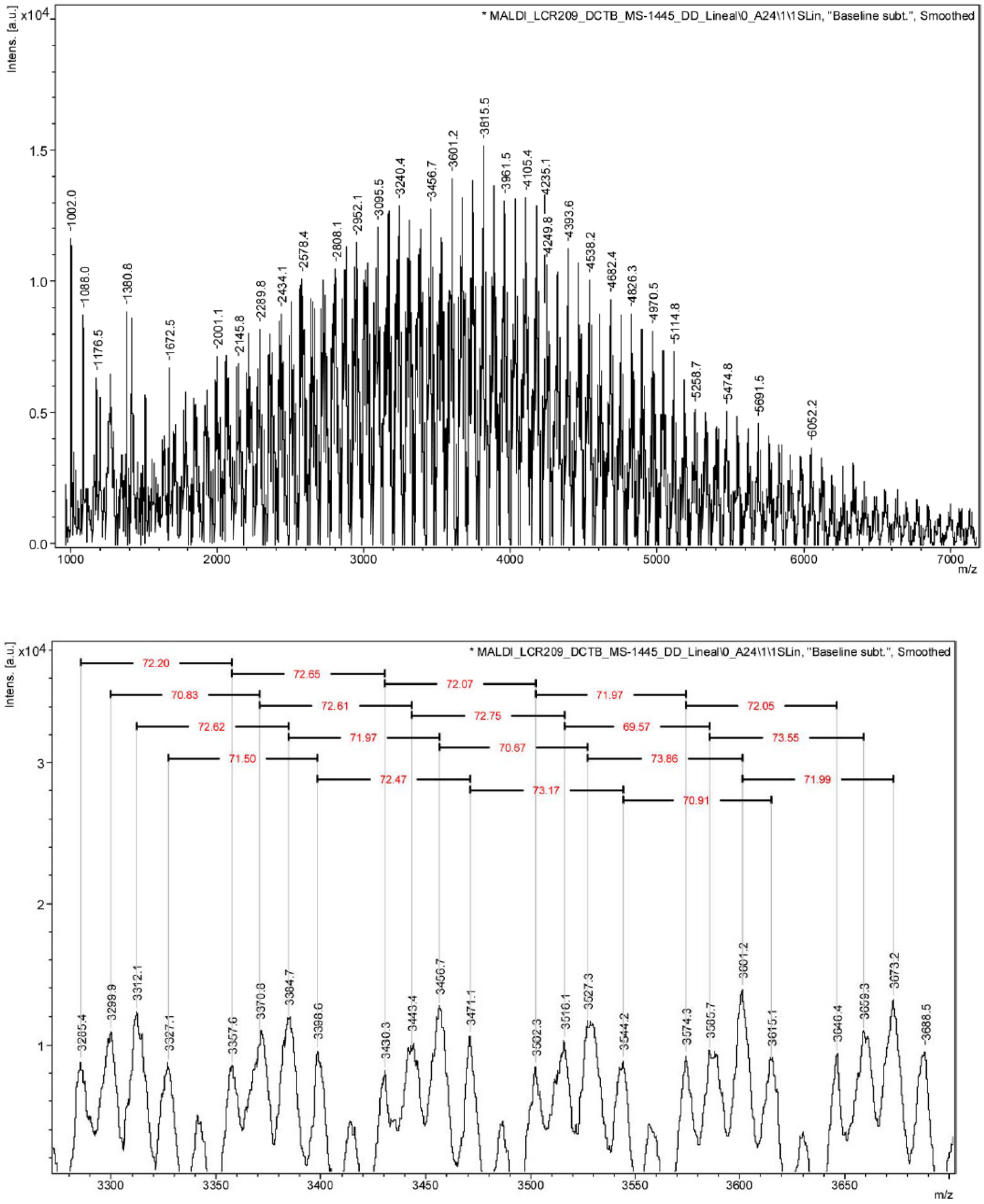

Figure S21. MALDI-TOF MS analysis of Compound **4.**

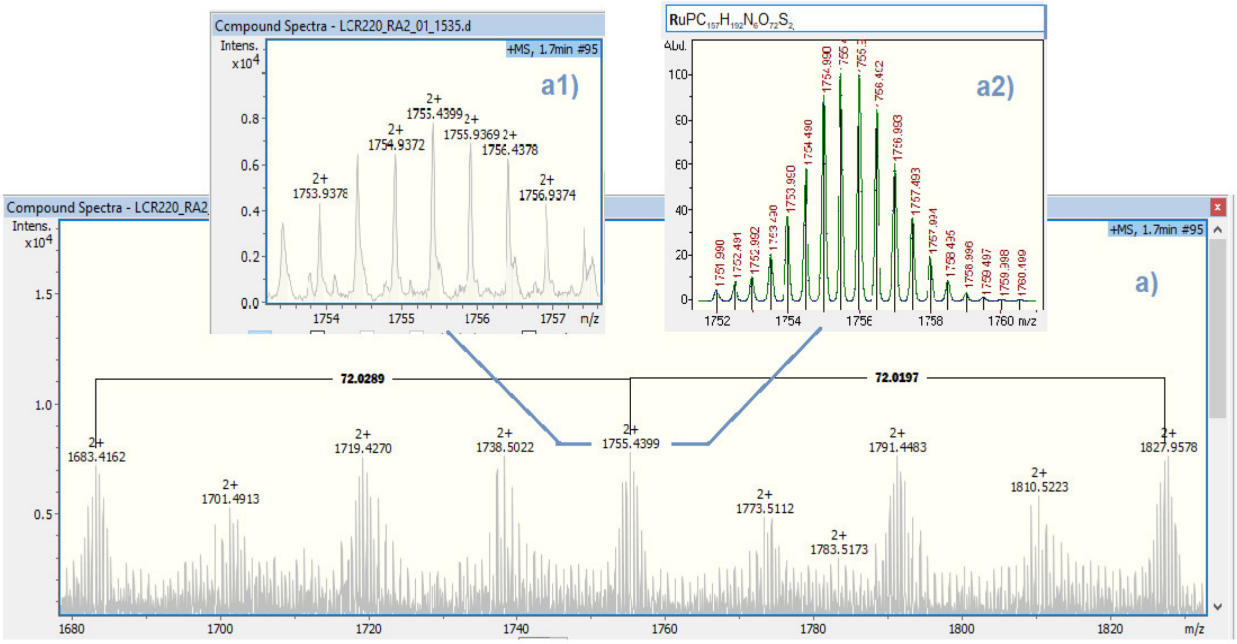

Figure S22. Partial HR mass spectrum obtained in the ESI positive mode of a methanolic solution of complex **1.** **a**) series of double charge ions attributed to [Ru(η^5-Cp)(P(C_6H_5)$_3$)(2,2′-bipy-4,4′-(PLA)$_{n=16;17;18}$-biotin)]$^{2+}$ structures spaced by 72.02 u; **a1**) the group of peaks at *m*/*z* 1755.44 assigned to [Ru(η^5-Cp)(P(C_6H_5)$_3$)(2,2′-bipy-4,4′-(PLA)$_{n=16;17;18}$-biotin)]$^{2+}$ exhibited a measured isotopic pattern as the calculated for the sum [$RuPC_{157}H_{192}N_6O_{72}S_2$]$^{2+}$ shown in **a2**).

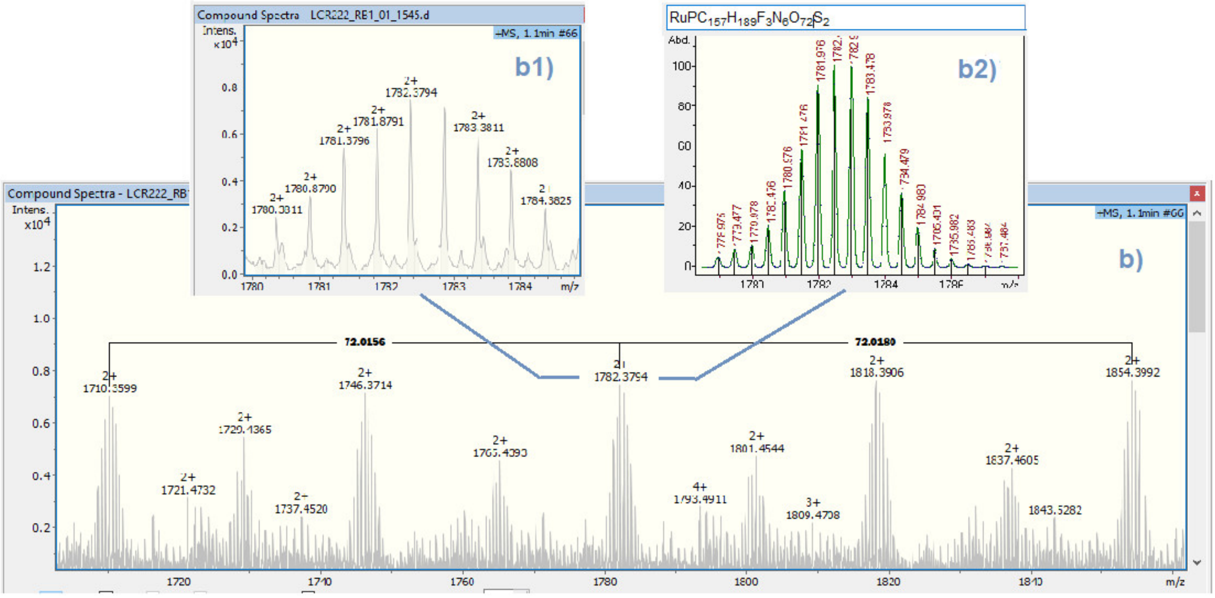

Figure S23. Partial HR mass spectrum obtained in the ESI positive mode of a methanolic solution of complex **2. b**) series of double charge ions attributed to $[Ru(\eta^5\text{-}Cp)(P(C_6H_4F)_3)(2,2'\text{-bipy-}4,4'\text{-}(PLA)_{n=16;17;18}\text{-biotin})]^{2+}$ structures spaced by 72.02 u; **b1**) the group of peaks at *m/z* 1782.47 assigned to $[Ru(\eta^5\text{-}Cp)(P(C_6H_4F)_3)(2,2'\text{-bipy-}4,4'\text{-}(PLA)_{n=16;17;18}\text{-biotin})]^{2+}$ exhibited a measured isotopic pattern as the calculated for the sum $[RuPC_{157}H_{189}F_3N_6O_{72}S_2]^{2+}$ shown in **b2**).

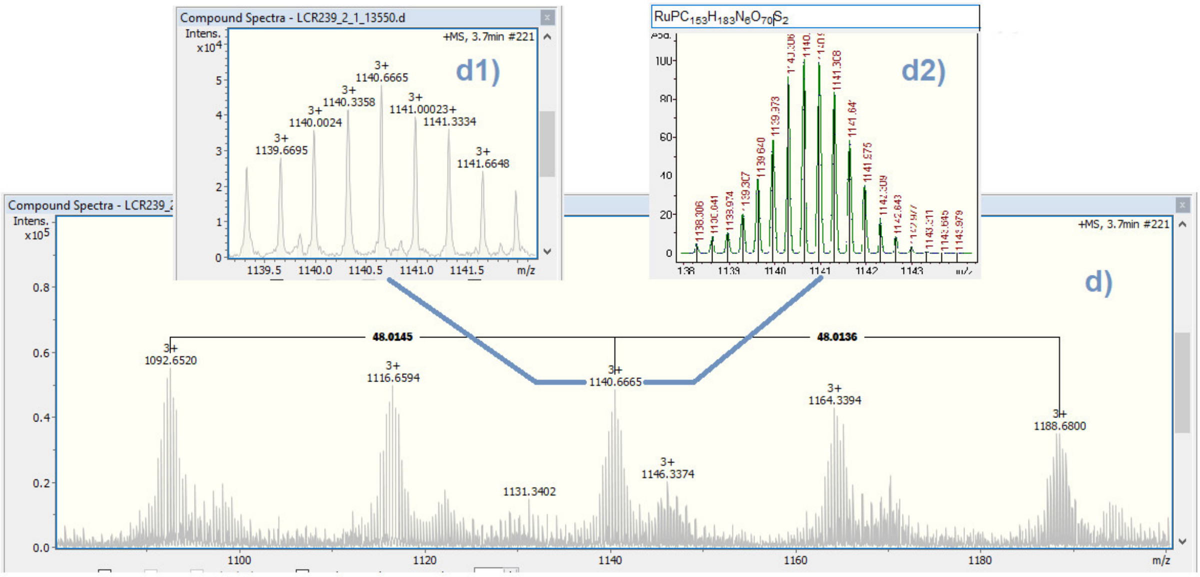

Figure S24. Partial HR mass spectrum obtained in the ESI positive mode of a methanolic solution of complex **3.** **d**) series of triple charge ions attributed to [Ru(η^5-Cp)(P($C_6H_4OCH_3$)$_3$(2,2′-bipy-4,4′-(PLA)$_{n=15;16;17}$)-biotin)]$^{3+}$ structures spaced by 48.01 u; **d1**) the group of peaks at *m*/*z* 1188.35 assigned to [Ru(η^5-Cp)[P(C_6H_4)$_3$(CHO)$_2$](2,2′-bipy-4,4′-(PLA)$_{n=16}$-biotin)]$^{3+}$ exhibited a measured isotopic pattern as the calculated for the sum [$RuPC_{153}H_{183}N_6O_{70}S_2$]$^{3+}$ shown in **d2**).

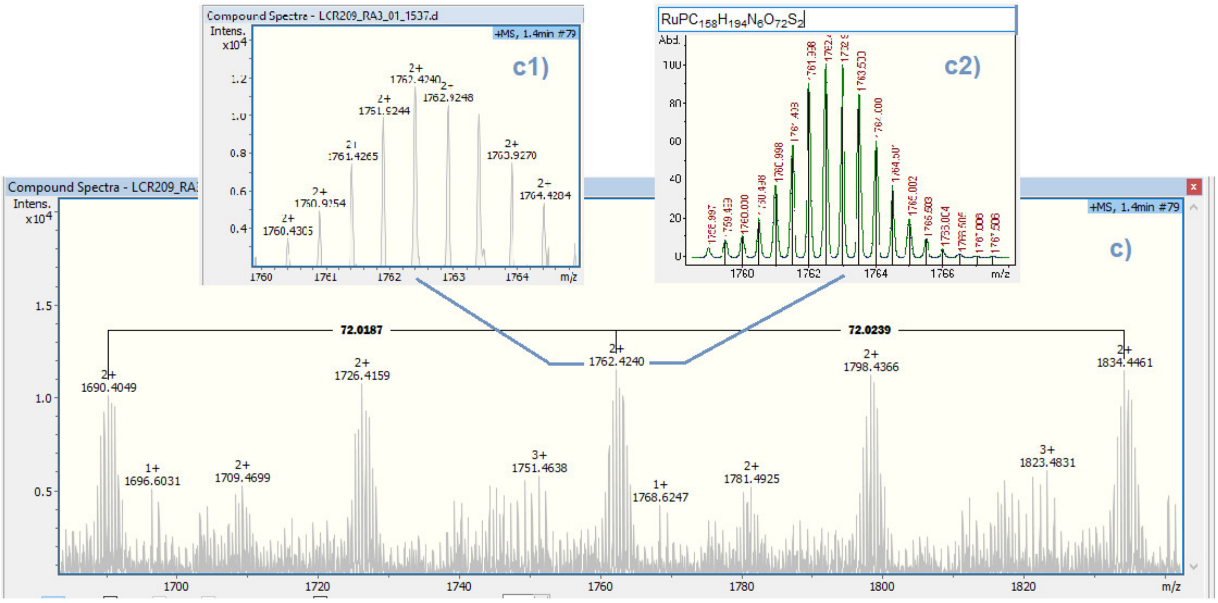

Figure S25. Partial HR mass spectrum obtained in the ESI positive mode of a methanolic solution of complex **4.** **c**) series of double charge ions attributed to [Ru(η^5-CpCH$_3$)(P(C$_6$H$_5$)$_3$)(2,2′-bipy-4,4′-(PLA)$_{n=16;17;18}$)-biotin)]$^{2+}$ structures spaced by 72.02 u; **c1**) the group of peaks at *m/z* 1762.42 assigned to [Ru(η^5-CpCH$_3$)(P(C$_6$H$_5$)$_3$)(2,2′-bipy-4,4′-(PLA)$_{n=16;17;18}$)-biotin)]$^{2+}$ exhibited a measured isotopic pattern as the calculated for the sum [$RuPC_{158}H_{194}N_6O_{72}S_2$]$^{2+}$ shown in **c2**).

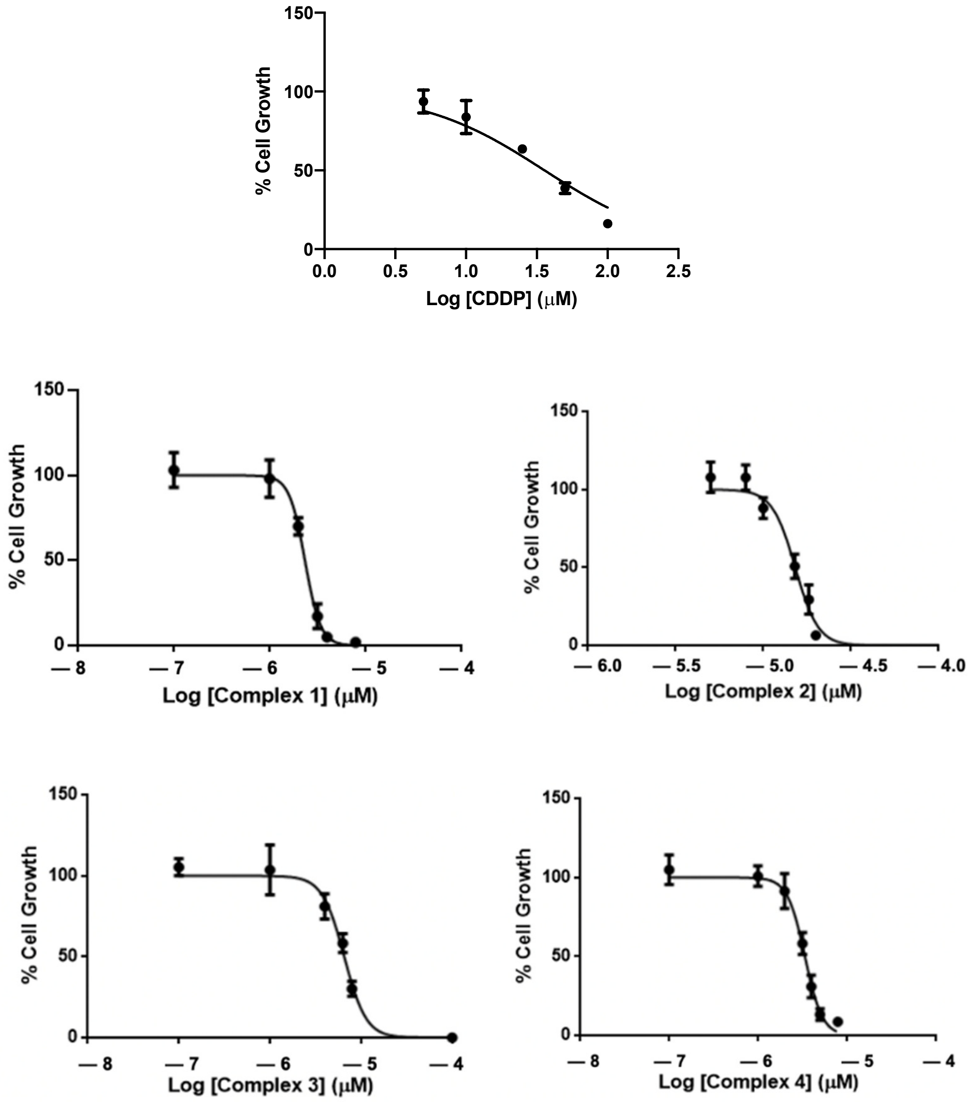

Figure S26. Dose-response curve of cell viability in MDA-MB-231 cell line for determination of IC_{50} by MTT (complexes **1**-**4**) or Sulforhodamine B (Cisplatin) after 48 h incubation.

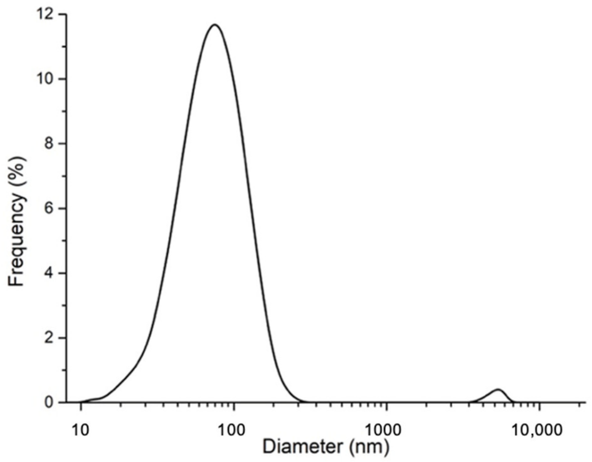

Figure S27. Hydrodynamic diameter distribution (obtained by DLS) of complex **1**.

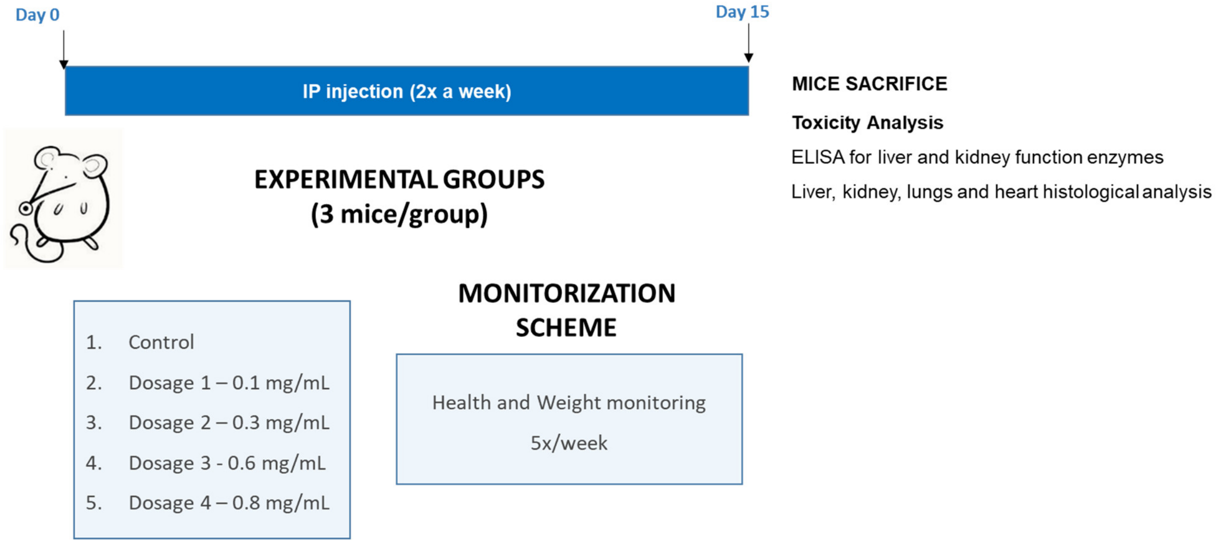

Figure S28. Experimental design of toxicology assay performed.

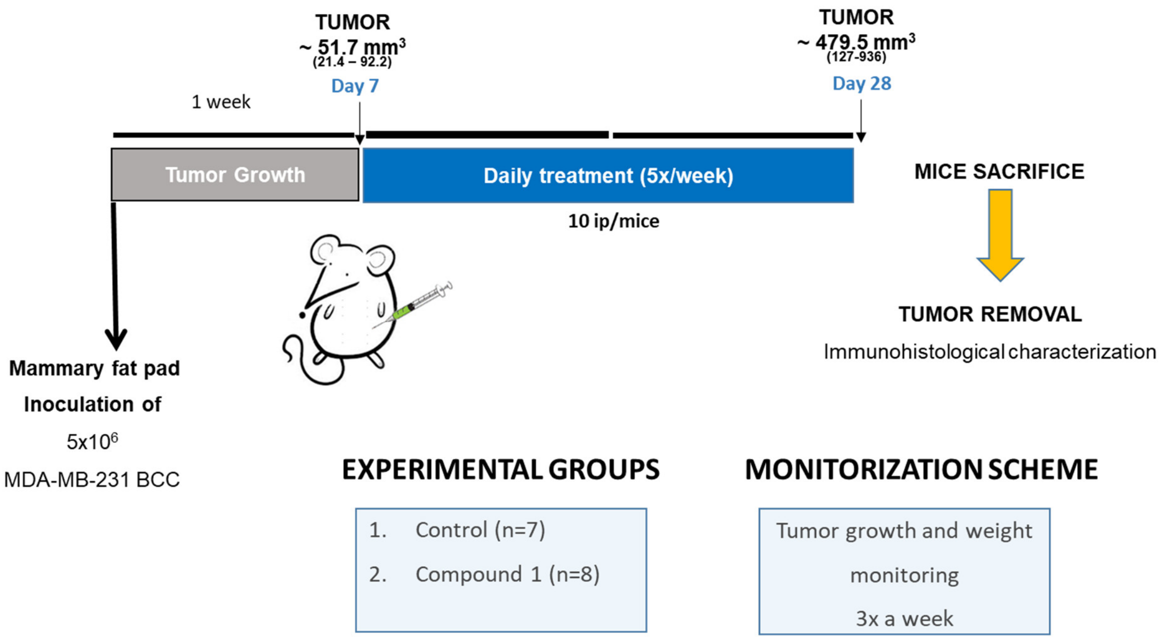

Figure S29. Experimental design of tumor growth assay.
